# RAG1 splicing mutation causes enhanced B cell differentiation and autoantibody production

**DOI:** 10.1172/jci.insight.148887

**Published:** 2021-10-08

**Authors:** Qing Min, Xin Meng, Qinhua Zhou, Ying Wang, Yaxuan Li, Nannan Lai, Ermeng Xiong, Wenjie Wang, Shoya Yasuda, Meiping Yu, Hai Zhang, Jinqiao Sun, Xiaochuan Wang, Ji-Yang Wang

**Affiliations:** 1 Department of Immunology, School of Basic Medical Sciences, Fudan University, Shanghai, China.; 2 Department of Clinical Immunology, Children’s Hospital of Fudan University, National Children’s Medical Center, Shanghai, China.; 3 School of Computing, Tokyo Institute of Technology, Yokohama, Japan.; 4 Department of Microbiology and Immunology, College of Basic Medical Sciences, Zhengzhou University, Zhengzhou, China.

**Keywords:** Autoimmunity, Immunology, Autoimmune diseases, Bone marrow differentiation, Immunoglobulins

## Abstract

Hypomorphic *RAG1* or *RAG2* mutations cause primary immunodeficiencies and can lead to autoimmunity, but the underlying mechanisms are elusive. We report here a patient carrying a c.116+2T>G homozygous splice site mutation in the first intron of *RAG1*, which led to aberrant splicing and greatly reduced RAG1 protein expression. B cell development was blocked at both the pro-B to pre-B transition and the pre-B to immature B cell differentiation step. The patient B cells had reduced B cell receptor repertoire diversity and decreased complementarity determining region 3 lengths. Despite B cell lymphopenia, the patient had abundant plasma cells in the BM and produced large quantities of IgM and IgG Abs, including autoantibodies. The proportion of naive B cells was reduced while the frequency of IgD^–^CD27^–^ double-negative (DN) B cells, which quickly differentiated into Ab-secreting plasma cells upon stimulation, was greatly increased. Immune phenotype analysis of 52 patients with primary immunodeficiency revealed a strong association of the increased proportion of DN B and memory B cells with decreased number and proportion of naive B cells. These results suggest that the lymphopenic environment triggered naive B cell differentiation into DN B and memory B cells, leading to increased Ab production.

## Graphical abstract

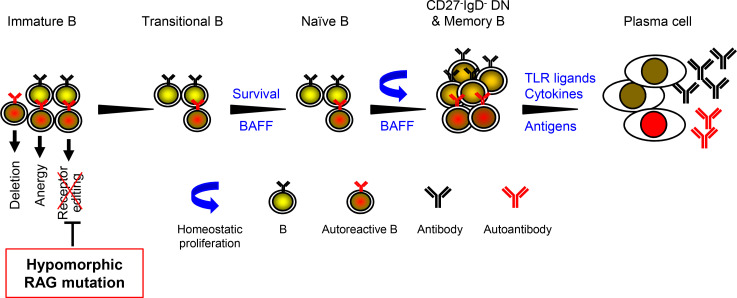

## Introduction

The vertebrate adaptive immune system relies on a diverse repertoire of antigen receptors on lymphocytes to recognize millions of possible antigens and generate specific Abs and effector T cells (1). The highly diversified repertoire is initially generated by somatic recombination of variable (*V*), diversity (*D*), and joining (*J*) gene segments (2–4). RAG1 and RAG2 proteins form a complex and initiate V(D)J recombination by introducing DNA double-strand breaks (DSBs) between the recombination signal sequences and the flanking *V*, *D*, or *J* gene segment. The resulting sealed hairpin coding ends and blunt signal ends are processed and joined by the nonhomologous end joining (NHEJ) repair pathway (4, 5). In addition to an essential role in the cleavage phase of V(D)J recombination, the RAG complex also has important functions in the joining phase. After the cleavage, the RAG complex remains associated with the DNA ends in a postcleavage complex that facilitates DNA end processing and ligation using the NHEJ machinery rather than other repair pathways, which helps prevent aberrant recombination events (5, 6). The joining of *V*, *D*, and *J* segments is usually imprecise and can generate nucleotide deletions and insertions by 3 mechanisms. Asymmetrical opening of the hairpin coding ends creates single-stranded extension and results in palindromic insertions (P nucleotides) (7). In addition, a few nucleotides can be deleted from the ends of the coding segments by an exonuclease before ligation. Furthermore, a variable number of nucleotides can be inserted at the *D*-*J* and *V*-*D* junctions by the lymphoid-specific terminal deoxynucleotidyl transferase (TdT), which randomly adds nontemplated nucleotides onto the coding ends (N nucleotides) (8).

Multiple V(D)J recombination events must occur during the genesis of each new lymphocyte (1). In humans, B cell development proceeds in an orderly fashion in the BM through several stages, including pro-B, pre-B, and finally IgM^+^ immature B cells (9). Pro-B cells upregulate RAG expression to initiate Ig heavy (IgH) chain *D* to *J* and then *V* to *DJ* joining to become pre-B cells. If a μ heavy chain protein can be expressed from the resulting *VDJCμ* mRNA, pre-B cells will express a pre-B cell receptor (pre-BCR) composed of 2 covalently linked μ heavy chains, 2 surrogate light chains, and 1 Igα/β chaperone/signal transduction module. Pre-B cells then undergo Ig κ or λ light chain gene recombination to differentiate into immature IgM^+^ B cells (9, 10).

*RAG1* or *RAG2* mutations in humans lead to a broad spectrum of clinical phenotypes depending on the nature of the defect and its quantitative and qualitative effects on V(D)J recombination. Complete loss-of-function mutations in *RAG1* or *RAG2* cause SCID with absence of mature T and B cells as well as all isotypes of Abs (11). Hypomorphic mutations in *RAG1* or *RAG2* with partial activity of RAG are associated with (a) Omenn syndrome (OS) with oligoclonal, activated, and anergic autologous T cells that infiltrate target tissues; hypereosinophilia; and high IgE levels (12); (b) atypical or leaky SCID (AS) with the presence of residual autologous T cells and without clinical manifestations of OS (6); and (c) delayed-onset combined immunodeficiency with granulomas or autoimmunity manifestations (CID-G/AI) (13). Some other phenotypes are also associated with *RAG1* mutations, including chronic CMV or EBV infections with expansion of γ/δ T cells (14, 15), idiopathic CD4^+^ T cell lymphopenia (16), common variable immunodeficiency (17), IgA deficiency (18), and other miscellaneous presentations (19).

The human RAG1 protein is composed of 1043 amino acids, and the catalytic core (amino acids 387–1011) contains a nonamer-binding domain, a dimerization and DNA-binding domain, a pre-RNase H and catalytic RNase H domain, 2 zinc-binding domains, and the carboxy terminal domain, which are all crucial for V(D)J recombination (4). Recently, a number of missense and frameshift mutations located in these domains have been reported to be associated with CID-G/AI (4). We report here what is likely the first splice site mutation in the *RAG1* gene in a patient with CID-G/AI. The patient exhibited impaired B cell development but enhanced B cell differentiation. Moreover, the patient had abundant plasma cells in the BM and produced large amounts of IgM and IgG Abs including autoantibodies. Detailed phenotypic analysis of B cells from 52 patients with primary immunodeficiency (PID) revealed a strong association of the increased frequency of IgD^–^CD27^–^ double-negative (DN) B and memory B cells with the reduced number and proportion of naive B cells. Moreover, these DN B cells were able to quickly differentiate into IgG-secreting plasma cells upon stimulation. These results suggest that the lymphopenic environment in patients with PID can trigger naive B cell differentiation into DN B and memory B cells, leading to elevated production of Abs, including autoantibodies.

## Results

### Clinical manifestation of the patient and identification of a homozygous splice site mutation in RAG1.

**Figure 1:**
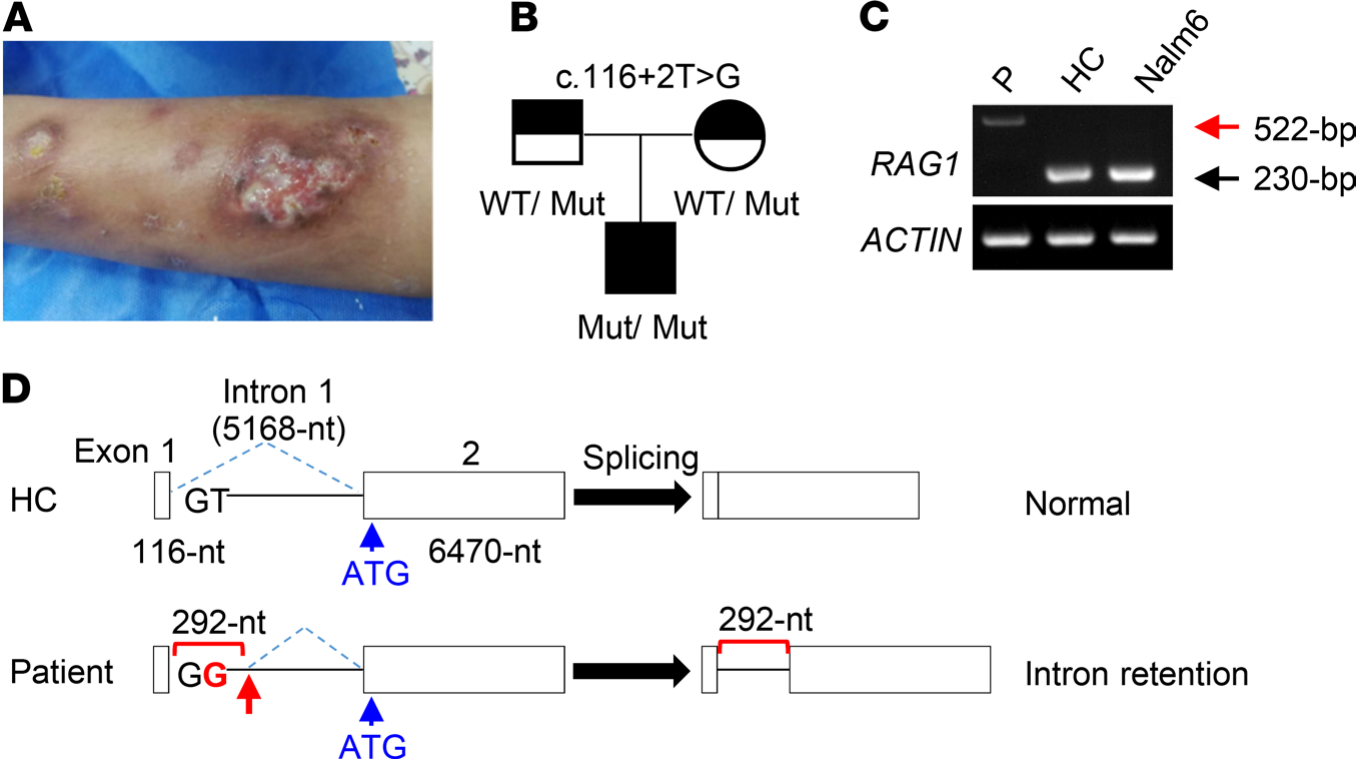
Clinical presentation and identification of a homozygous *RAG1* splice site mutation in a patient with CID-G/AI. (**A**) The skin lesions on the lower limbs of the patient. (**B**) Pedigree of the family. (**C**) Reverse transcription PCR analysis of *RAG1* mRNA splicing and expression. First strand cDNA from BMMNCs of the patient (P) and HC and from human pre-B cell line Nalm6 was amplified as described in Methods. As a positive control, the first strand cDNA was also amplified for *ACTIN* expression. (**D**) Schematic illustration of the genomic structure of WT (upper) and mutant (lower) *RAG1* genes. The location of the authentic translation initiation site (ATG) is shown by a blue arrow. The mutant base (G) is shown in red, and the cryptic splice donor site is shown by a red arrow.

The patient was the first child of nonconsanguineous Chinese parents, born healthy after a normal gestation. There was no history of PID in his family. Initially he was in a relatively healthy condition, except for recurrent mild upper respiratory infections and being hospitalized twice due to varicella infection and pneumonia at 2.5 and 4 years of age, respectively. At 5.5 years of age, he developed frequent epistaxis with low platelet count (5 × 10^9^ to 251 × 10^9^/L), multiple lymphadenopathy, and mild hepatosplenomegaly. Pathological biopsy of inguinal lymph nodes revealed granulomatous inflammation accompanied by necrosis. He also suffered from recurrent bacterial, viral, and parasitic infections as well as tuberculosis infections of several organs, including lung, liver, and testis. He received intravenous Ig (IVIG) twice, at 5.5 and 6.5 years of age. The platelet count recovered after the first IVIG infusion. At age 6, the patient developed spontaneous tissue destruction that resulted in erosion of his nasal septum and paravertebral and retroperitoneal soft tissue swelling, as well as swelling and ulceration in the lower limbs and face (Figure 1A). Biopsy of a skin mass suggested local fibrovascular hyperplasia accompanied by infiltration of a large number of acute and chronic inflammatory cells, mainly T cells, and biopsy of skin lesions demonstrated vasculitis with neutrophilic and granulomatous inflammation.

A detailed immunological profile of the patient was performed 3 times, at 6, 7, and 8 years of age, and revealed age-dependent rapid decreases in the numbers of B and T cells and a reduction of NK cells, especially between ages 7 and 8 (Table 1). Despite B cell lymphopenia, the levels of serum IgM, IgA, and IgG (predominantly IgG_1_) were elevated while the IgE level was normal (Table 1 and Supplemental Table 1; supplemental material available online with this article; https://doi.org/10.1172/jci.insight.148887DS1), and ANA, pANCA, and PR3-proteinase were also positive (Table 1). The patient had mild anemia with hemoglobin range from 92 to 118 g/L. Although Coombs test was positive in the patient, the indirect bilirubin and the lactate dehydrogenase were both normal. Due to the typical immunological phenotype, recurrent infection, granulomatous formation, and autoimmunity, the patient was diagnosed at 8 years old with CID-G/AI. Although the patient received intensive antimicrobial treatment and prophylaxis and glucocorticosteroids were administered due to the severe autoimmunity, his clinical condition repeatedly worsened. As the clinical condition could not be controlled by conservative treatment methods, the patient underwent cord blood stem cell transplantation at age 8. A myeloablative regimen was used, which included fludarabine, cyclophosphamide, and busulfan. He had dyspnea 8 days after hematopoietic stem cell transplantation and was suspected to have engraftment syndrome. As the condition deteriorated, he was sent to the pediatric intensive care unit. One month later, he died of multiple organ failure and multiple infections. Chimerism at days 14 and 30 after the transplantation reached 100%.

Whole-exome sequencing (WES) analysis identified a potentially novel c.116+2T>G (IVS1+2T>G) homozygous splice site mutation in the *RAG1* gene of the patient (Figure 1B). His parents were both heterozygous carriers of the same mutation (Figure 1B). To characterize the possible defect in *RAG1* RNA splicing in the patient, we isolated total RNA from BM mononuclear cells (BMMNCs) of the patient and an age-matched healthy control (HC) and from the Nalm6 human pre-B cell line. Total RNA was first treated with DNase I to eliminate any contaminating genomic DNA and then used to synthesize the first strand cDNA, which was then amplified by PCR. As shown in Figure 1C, a 522 bp band was amplified in the patient sample (red arrow), in contrast to the correct 230 bp band in HC and Nalm6 samples.

To clarify the nature of the larger mRNA the patient expressed, we sequenced the 522 bp as well as the 230 bp bands. As expected, the 230 bp band corresponded to the correctly spliced *RAG1* mRNA. In contrast, the 522 bp band contained an additional 292 bp derived from the first intron (Figure 1D), which included 12 extra ATG codons (Supplemental Figure 1). Eleven (underlined in red) out of the 12 ATGs were followed by premature termination codons (PTCs, squared), and only the second to the last extra ATG (underlined in black) was in frame with the authentic ATG (shown in blue). The multiple upstream ATGs with PTCs likely interfere with the translation of RAG1 protein from its authentic ATG in exon 2 and also may cause nonsense-mediated mRNA decay (NMD), a quality control mechanism in eukaryotes that surveys newly synthesized mRNAs and degrades those that harbor a PTC (20, 21).

### Aberrant splicing reduced RAG1 protein expression.

**Figure 2:**
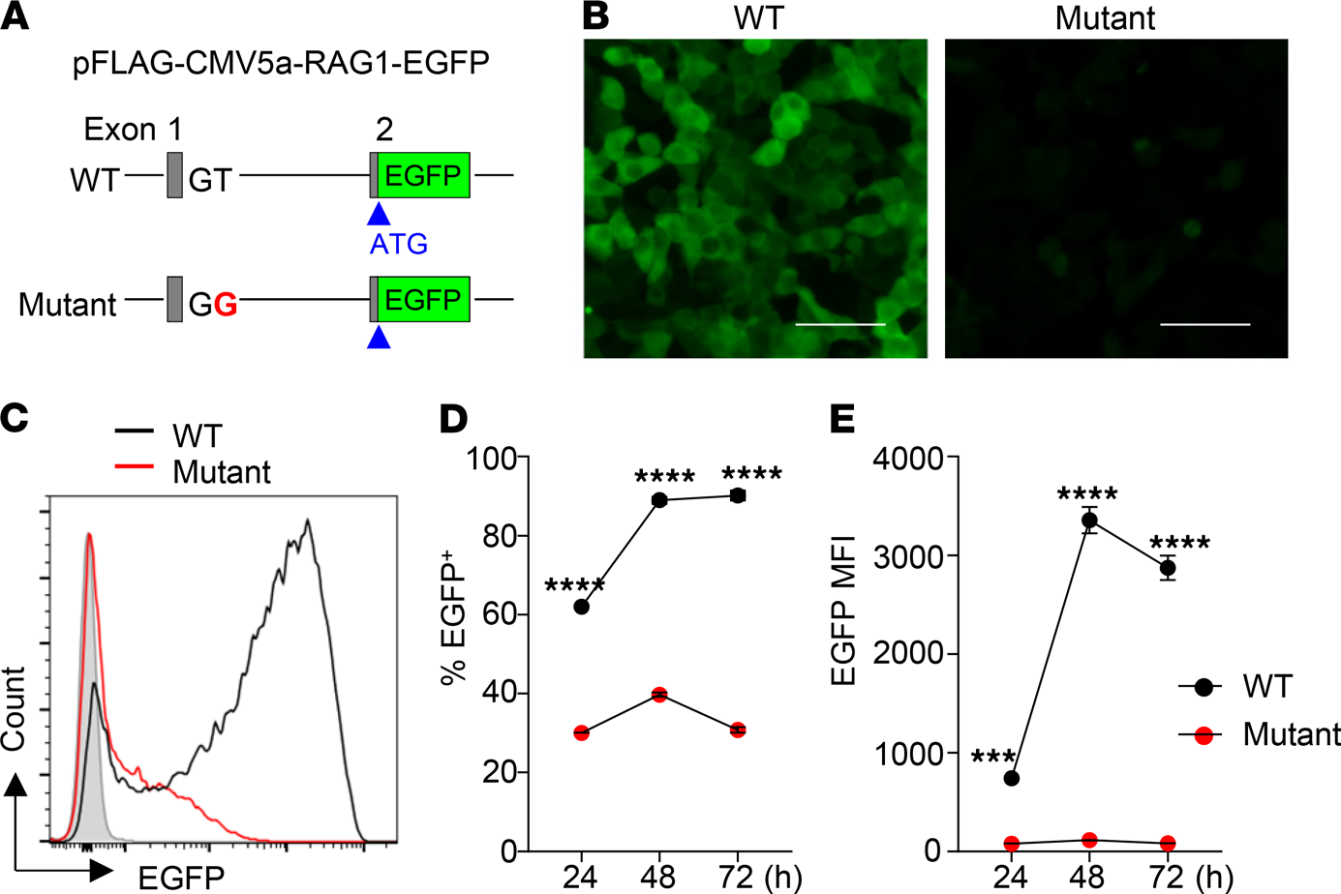
Analysis of RAG1 expression using a sensitive EGFP reporter assay. (**A**) Schematic structure of the RAG1-EGFP reporter vector. The WT or mutant RAG1-EGFP reporter vector was transfected into 293T cells and analyzed for EGFP expression by fluorescence microscopy and flow cytometry at 24 hours, 48 hours, and 72 hours after the transfection. (**B**) Representative images at 48 hours. Scale bar: 200 μm. (**C**) Representative FACS profiles at 48 hours. (**D**) Percentage of EGFP^+^ cells. (**E**) MFI of EGFP. Mean ± SD of 3 independent experiments are shown. Statistical analyses were performed using Student’s *t* test. ***P < 0.001; ****P < 0.0001.

To explore how the aberrant splicing of *RAG1* mRNA affected RAG1 protein expression, we attempted to perform immunoblot analysis using BM cells from the patient and an age-matched HC. However, we were unable to detect a RAG1 band in both the patient and HC possibly because of the low numbers of the pro- and pre-B cells contained in the BM. To estimate the RAG1 levels in the patient relative to HC, we then constructed a sensitive EGFP reporter vector, in which a *RAG1* genomic fragment containing exon 1, intron 1, and part of exon 2 (including the authentic translation initiation site) derived from HC or the patient was inserted upstream of and in frame with *EGFP* (Figure 2A). We then transfected the RAG1-EGFP reporter vector into 293T cells and monitored the expression of EGFP at different time points by using fluorescence microscopy and flow cytometry. As shown in Figure 2B, EGFP was highly expressed in 293T cells transfected with WT RAG1-EGFP vector. In striking contrast, EGFP fluorescence was only faintly detectable in 293T cells transfected with the mutant RAG1-EGFP vector (Figure 2B). Consistently, FACS analysis revealed that 293T cells transfected with the mutant RAG1-EGFP had a much lower proportion of EGFP^+^ cells (Figure 2, C and D), and the mean fluorescence intensity (MFI) was also greatly reduced compared with those transfected with WT RAG1-EGFP (Figure 2E). These results suggest that the splice site mutation greatly reduced RAG1 protein expression.

### Impaired B and T cell development and enhanced B cell differentiation in the patient.

**Figure 3:**
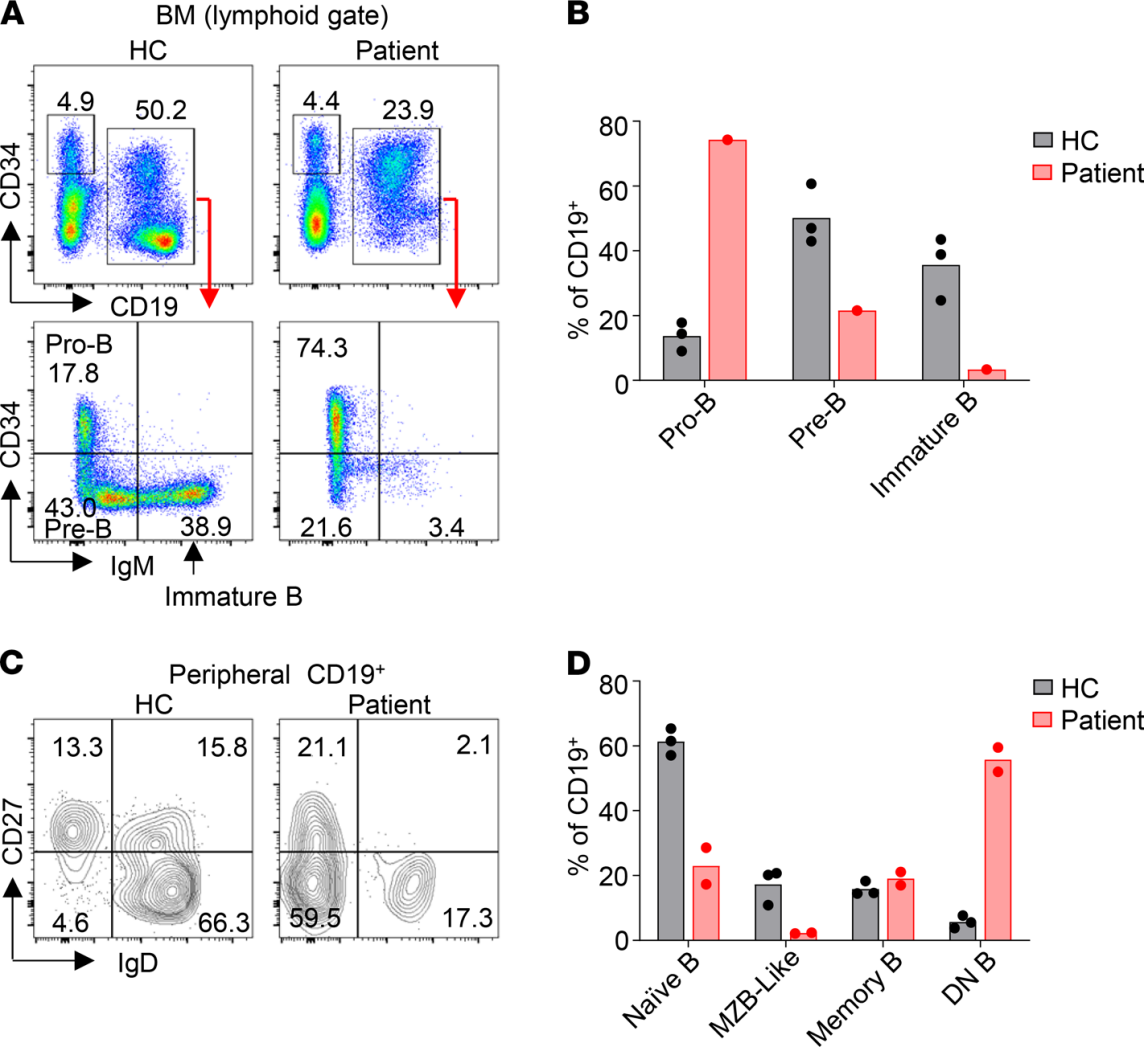
Impaired B cell development and maturation in the patient. (**A** and **B**) Impaired B cell development in the patient. (**A**) FACS analysis of pro-B (CD19^+^CD34^+^IgM^–^), pre-B (CD19^+^CD34^–^IgM^–^), and immature B (CD19^+^CD34^–^IgM^+^) cells in the BM of the patient and HC. (**B**) Mean of the results in **A** obtained with the patient and 3 HCs. (**C** and **D**) Enhanced B cell differentiation. (**C**) FACS profiles showing the peripheral blood naive B (IgD^+^CD27^–^), MZB-like (IgD^+^CD27^+^), memory B (IgD^–^CD27^+^) and DN B (IgD^–^CD27^–^) population. (**D**) Mean of the results in **C** obtained with the patient (2 independent experiments) and 3 HCs is shown.

We next analyzed B cell development in the BM and B and T cell compartments in the peripheral blood. The proportion of CD19^+^ cells in patient BM was approximately half of that in HC (Figure 3A). Among the CD19^+^ cells, the CD34^+^IgM^–^ pro-B population was greatly increased in the patient (74.3% vs. 13.8 ± 4.4% in HC, mean ± SD) while the CD34^–^IgM^–^ pre-B population was decreased (21.6% vs. 50.2 ± 9.3% in HC) (Figure 3B), suggesting a block in B cell development at the pro-B to pre-B transition. Moreover, the CD34^–^IgM^+^ immature B cell population was reduced to 3.4% in the patient compared with 35.7 ± 9.8% in HC (Figure 3B) and expressed low levels of cell surface IgM, demonstrating that pre-B to immature B cell differentiation was severely impaired in the patient. In addition, the proportions of both naive B (CD19^+^IgD^+^CD27^–^) and marginal zone B–like (MZB-like) (CD19^+^IgD^+^CD27^+^) cells were decreased in the patient compared with HC (Figure 3, C and D). Notably, more than 50% of the peripheral blood B cells in the patient had a DN (CD19^+^IgD^–^CD27^–^) phenotype (Figure 3D), a population previously found to be increased in patients with autoimmune diseases and in elderly individuals (22). The DN B cells in the patient expressed higher levels of CD19 than did those in the HC (Supplemental Figure 2).

The patient had a greatly reduced number of lymphocytes in the peripheral blood (Table 1). FACS analysis revealed a dramatic decrease of naive T cells (CD45RA^+^CD27^+^), especially the CD4^+^ subset (Table 2). In T cell lymphopenic hosts, compensatory homeostatic proliferation can result in an increased proportion of peripheral T cells with an activated phenotype (6, 23). Consistently, central memory (CD45RA^–^CD27^+^) and effector memory (CD45RA^–^CD27^–^) T cells were both increased in the patient (Table 2). Notably, 23.1% of the CD3^+^ T cells in the patient were DN for CD4 and CD8 expression (Table 2), and almost all of them expressed a γ/δ T cell receptor. The proportion of γ/δ T cells among the CD3^+^ population was also higher in the patient than in HC (Table 2, bottom line). For the analysis of T cells in the patient, the data of age-matched HC were based on reference values for peripheral lymphocyte subsets of healthy children in China (24). These results indicate that T cell development and differentiation were also strongly affected in the patient. Consistent with previous findings in lymphopenic patients, the patient serum contained an elevated level of the B cell–activating factor (BAFF) compared with HC (Supplemental Figure 3).

### Reduced IgH repertoire diversity in the BM IgM and peripheral IgM and IgG in the patient.

**Figure 4:**
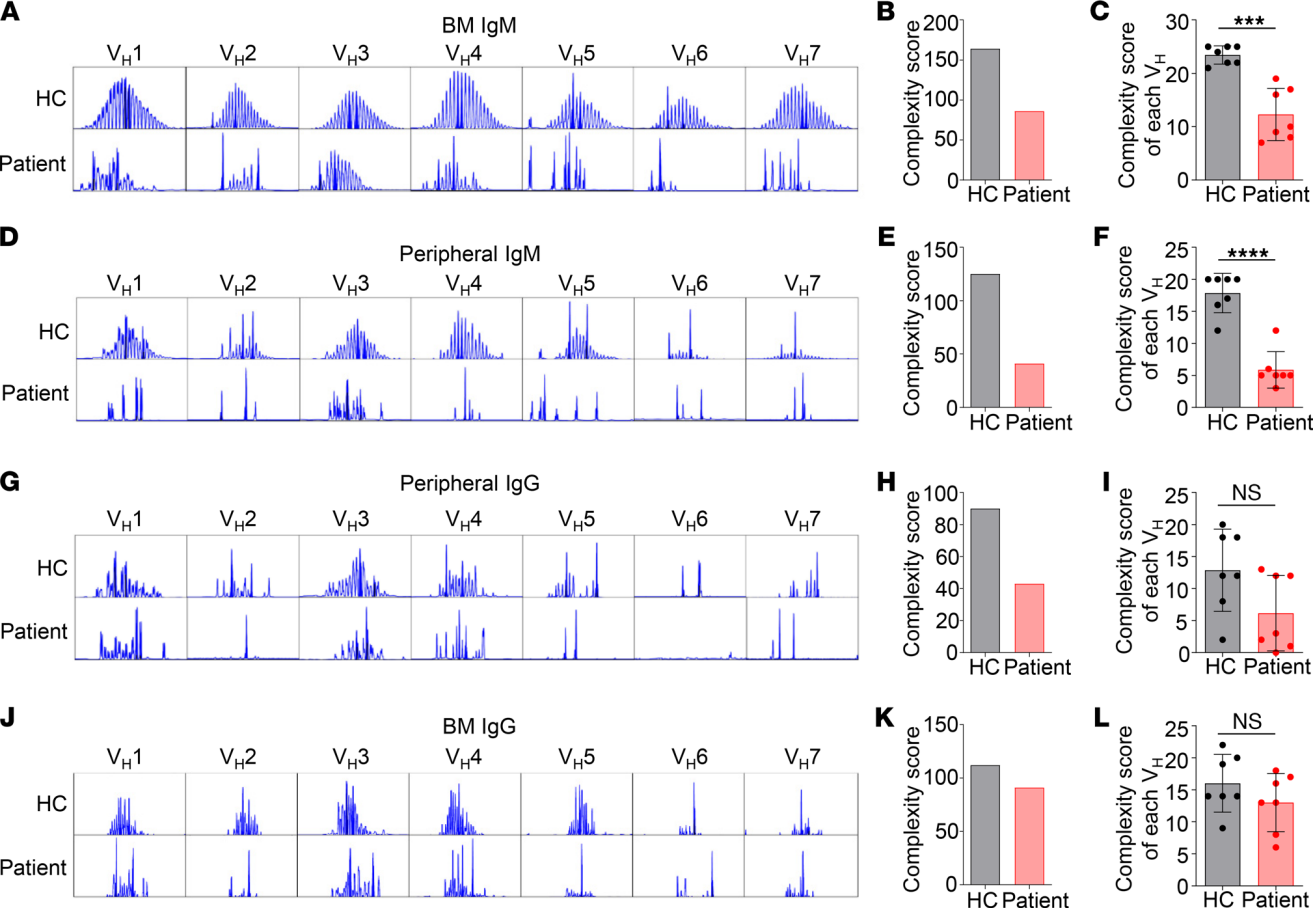
Reduced *IgH* diversity in the patient BM IgM and peripheral blood IgM and IgG relative to HC. *IgH* CDR3 profiles for each of the 7 *V_H_* families in BM IgM (**A**), peripheral IgM (**D**), peripheral IgG (**G**), and BM IgG (**J**). The *x* axis indicates CDR3 length, and the *y* axis displays fluorescence intensity. (**B**, **E**, **H**, and **K**) *IgH* repertoire diversity expressed as the sum of the number of different CDR3 lengths. (**C**, **F**, **I**, and **L**) Average number of CDR3 lengths for each of the *V_H_* genes (mean ± SD). Statistical analyses were performed using Student’s *t* test. ***P < 0.001; ****P < 0.0001.

To investigate the impact of the decreased RAG1 expression on Ig repertoire diversity, we first analyzed complementarity determining region 3 (CDR3) length distributions in each of the 7 heavy chain variable (*V_H_*) Ig gene families (*V_H_1*–*V_H_7*), for the IgM and IgG isotypes in both the BM and peripheral blood. The BM IgM of HC showed a symmetrical, normal distribution of CDR3 lengths (clonal peaks) for each of the *V_H_* families (Figure 4A), indicating a diversified *IgH* repertoire composed of various CDR3 lengths. In contrast, the patient BM IgM had a non-normal distribution for most *V_H_* families except for *V_H_3*, the largest human *V_H_* family, which had a close-to-normal distribution (Figure 4A). The sum of the number of clonal peaks, which correlated with the *IgH* repertoire diversity (25, 26), was calculated as a complexity score. In the BM, the combined complexity score of *V_H_1*–*V_H_7* families in the patient was only approximately half of that in HC (Figure 4B). The complexity score of individual *V_H_* genes was also significantly reduced in patient BM cells compared with HC (Figure 4C). These results demonstrate that IgM repertoire diversity is greatly reduced in the patient BM.

The number of clonal peaks for the IgM isotype in the peripheral blood (Figure 4, D–F) was generally lower than in the BM (Figure 4, A and B) both in the patient and HC, possibly because of antigen selection that occurs during B cell maturation and differentiation. Again, the patient’s peripheral IgM had fewer clones for most *V_H_* families than HC (Figure 4D). Both the total complexity score (Figure 4E) and the complexity score for each *V_H_* family (Figure 4F) were reduced in the patient.

The clonal peaks for peripheral IgG were also decreased in the patient compared with HC, though the difference did not reach statistical significance (Figure 4, G–I). However, *V_H_2*, *V_H_5*, *V_H_6*, and *V_H_7* families of IgG isotype contained only few clones in the patient (Figure 4G). These data demonstrate that *IgH* repertoire diversity was reduced in the patient both during B cell development in the BM and after B cell maturation and differentiation in the periphery.

We further compared the *V_H_* gene clonality in the BM IgG between the patient and HC. Despite the decreased diversity in the peripheral IgM and IgG in the patient compared with HC (Figure 4, E and H), the clonality in the BM IgG was quite similar between the patient and HC (Figure 4, J–L). Remarkably, the complexity score in the patient BM IgG (Figure 4K) was 2.2 and 2.1 times higher than that in the patient peripheral IgM (Figure 4E) and IgG (Figure 4H), respectively, in contrast to a similar complexity score found among BM IgG and peripheral IgM and IgG in HC.

### Increased IgH repertoire diversity in the patient BM IgG relative to the peripheral blood.

**Figure 5:**
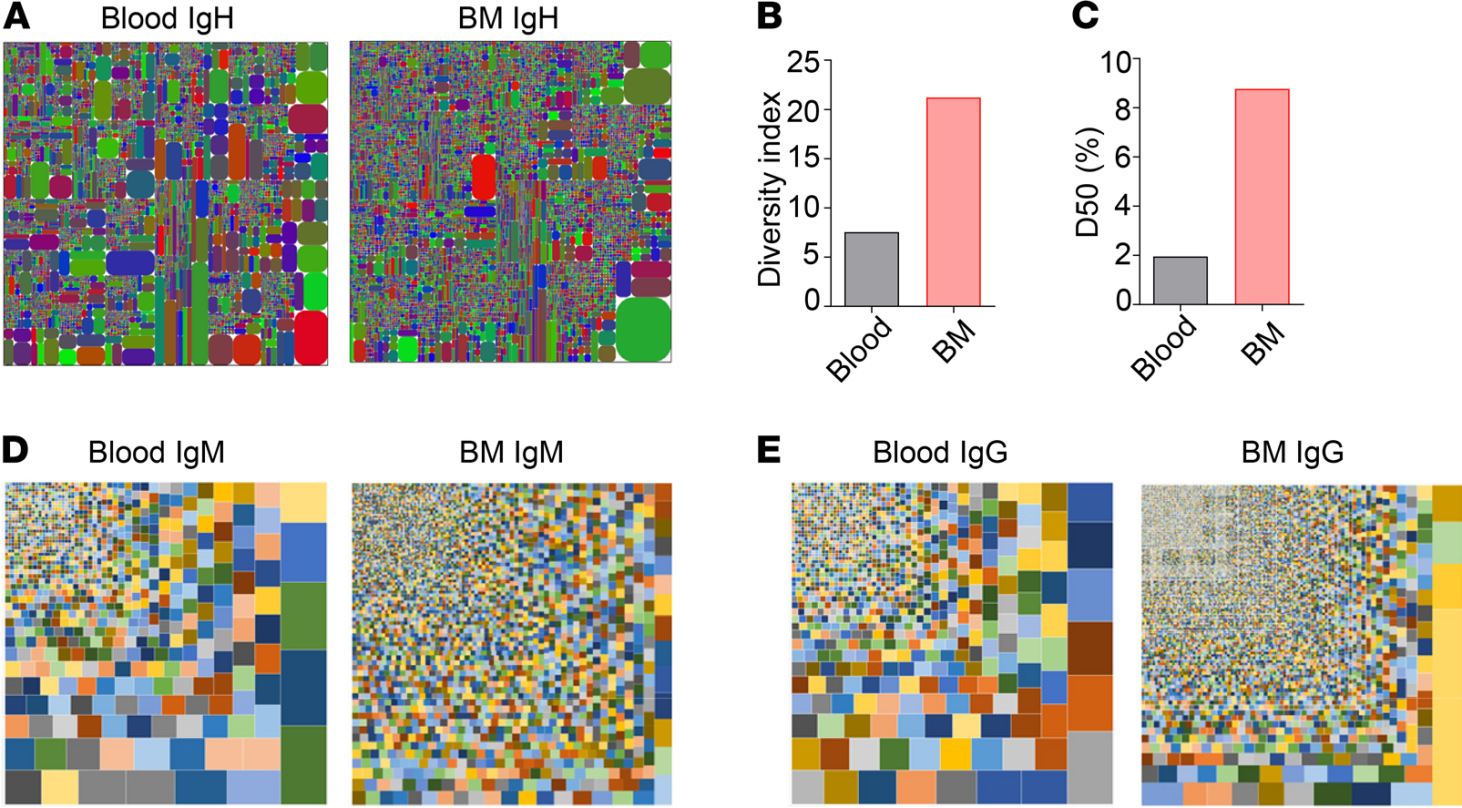
Analysis of the patient peripheral and BM *IgH* repertoire using NGS. (**A**) Tree maps showing the clonality and diversity of the peripheral blood and BM *IgH* transcripts. Each rectangle represents a unique CDR3 nucleotide sequence, and the size of the rectangle represents the relative frequency of an individual CDR3 sequence. (**B** and **C**) The diversity index (**B**) and D50 (**C**) of the patient peripheral and BM *IgH* CDR3 sequences. (**D** and **E**) Tree maps showing the clonality and diversity of the peripheral and BM *IgM* (**D**) or *IgG* (**E**) transcripts.

To further verify the increased IgG repertoire diversity in the patient BM, we performed next generation sequencing (NGS) of the patient BM and peripheral *IgH* transcripts. As shown in Figure 5A, tree maps revealed increased CDR3 clonality in the patient BM as compared with his peripheral blood. Consistently, diversity index plot showed increased diversity (Figure 5B) and higher D50 value (represents the number of the most dominant unique CDR3s among all unique CDR3s that make up 50% of the total effective reads, Figure 5C) in the BM relative to the peripheral blood. Among the 149,739 BM IgG CDR3 sequences, only 8.5% (12,738 sequences) were unique. This observation suggests that the patient BM IgG^+^ cells had undergone substantial clonal expansion. We further compared CDR3 nucleotide sequences of IgM and IgG from the peripheral blood and BM and found that for both isotypes the BM had greater diversity than did the blood (Figure 5, D and E). Consistently, tree maps of CDR3 amino acid sequences also supported increased clonality and diversity for both IgM and IgG from the patient BM relative to the blood (Supplemental Figure 4, A–C).

NGS also revealed increased frequency of IgG transcripts in the patient BM relative to the peripheral blood while the frequency of IgM transcripts was similar (Supplemental Figure 4E). Unexpectedly, the patient had a relatively high frequency of IgA transcripts (Supplemental Figure 4E), and again the BM had greater diversity than the blood (Supplemental Figure 4D). In addition, both the *V_H_* and *J_H_* genes in the patient BM contained more point mutations than those in the peripheral blood (Supplemental Figure 4F).

A comparison of the peripheral and BM IgG CDR3 sequences revealed that 7.457% of the peripheral CDR3 sequences were also found in the BM (Table 3), suggesting that these peripheral and BM IgG^+^ cells shared the same origin and that the BM IgG^+^ cells were derived from the peripheral IgG^+^ cells. Notably, 7.457% of the peripheral IgG CDR3 sequences corresponded to only 1.413% of the BM IgG CDR3 (Table 3), consistent with the finding that the patient BM contained a larger *IgH* repertoire than the peripheral blood (Figure 5). These results collectively suggest that the patient peripheral B cells differentiated into IgG^+^ cells that accumulated in the BM.

### Reduced CDR3 lengths in patient B cells.

**Figure 6:**
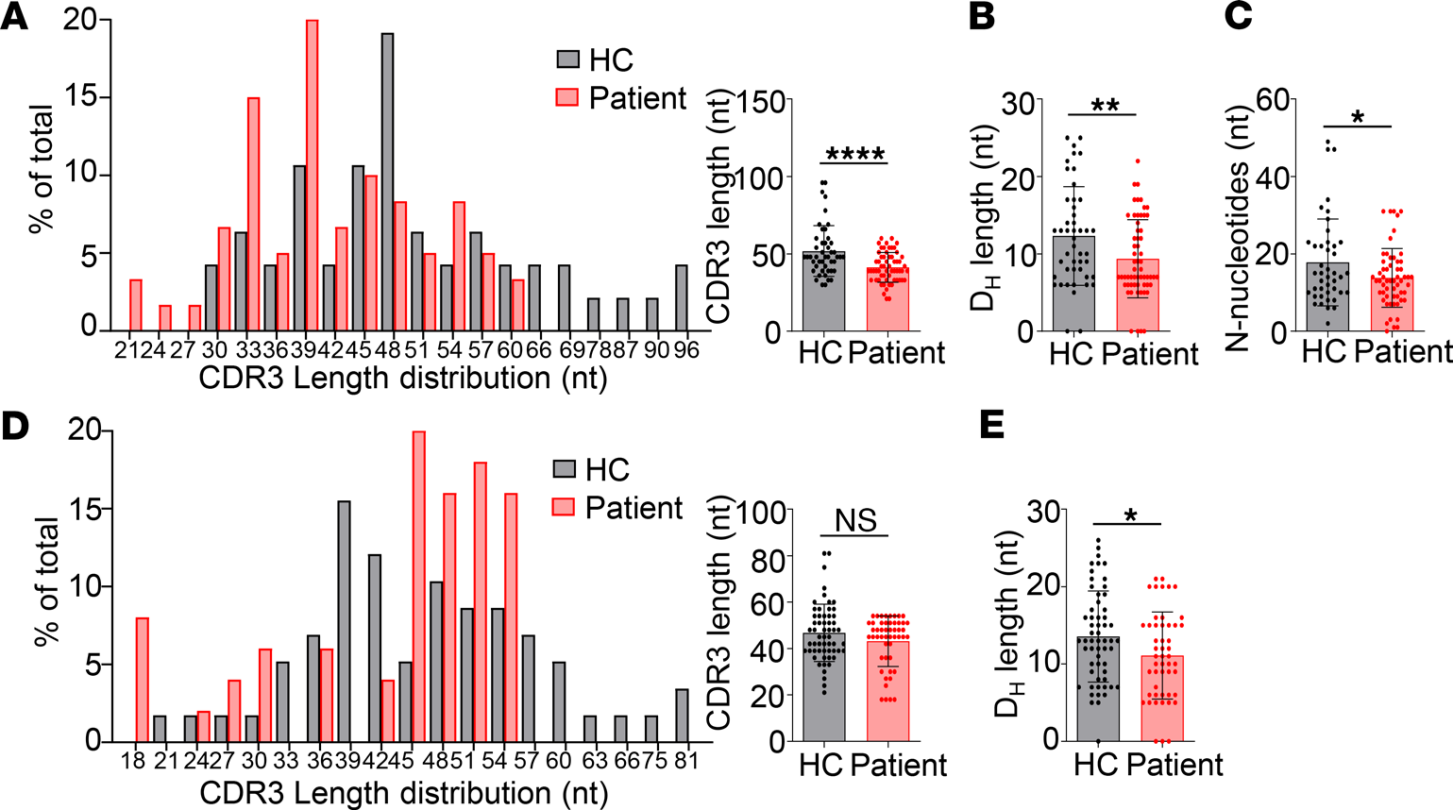
Decreased CDR3 lengths in the patient. (**A**–**C**) IgH-CDR3 profiles of the BM IgM isotype. (**A**) CDR3 length distribution and average CDR3 length (mean ± SD) of an HC and the patient. (**B**) Average length of the *D_H_* segments. Mean ± SD is shown. (**C**) Reduced N nucleotide addition in the patient (mean ± SD). (**D** and **E**) IgH-CDR3 profiles of peripheral IgM isotype. (**D**) CDR3 length distribution and average CDR3 length (mean ± SD). (**E**) Average length of the *D_H_* segments. Mean ± SD is shown. Statistical analyses were performed using Student’s *t* test. *P < 0.05; **P < 0.01; ****P < 0.0001.

The *IgH* CDR3 encompasses the tail of the *V_H_* gene, the entire *D_H_* segment, and the head of the *J_H_* gene, as well as *V*-*D* and *D*-*J* junctional sequences. These *V-D* and *D-J* junctions are formed by P nucleotide insertion, exonuclease-induced nucleotide deletion, and TdT-mediated N nucleotide addition (8). We amplified and sequenced *IgH* CDR3 from cDNAs of the BM and peripheral blood. About 90% and 95% of the sequences derived from the BM and peripheral blood were productive rearrangements, respectively. Analysis with the IMGT database revealed that CDR3 segments in the patient BM were significantly shorter than in HC (Figure 6A). Further analysis revealed that the shorter CDR3 was mainly due to decreased length of *D_H_* segments (Figure 6B) and fewer N nucleotides (Figure 6C). Since the lengths of the germline-encoded *D_H_* genes are variable, ranging from 11 bp to 37 bp, one possibility for the decreased length of *D_H_* segments in the patient is the preferential use of shorter *D_H_* segments. However, we found that the germline *D_H_* genes the patient and HC used had similar average lengths (Supplemental Figure 5A) and that the shorter *D_H_* length in the patient was due to increased exonuclease nibbling of *D_H_* segments (Supplemental Figure 5B). Although not statistically significant, the *D_H_* segments of the patient had more nucleotide deletions at both the 5′- and 3′-ends than did those of HC (Supplemental Figure 5C), suggesting that increased nibbling of the *D_H_* gene segments occurred during both *D-J* and *V* to *DJ* joining in the patient. P nucleotides were not different between the patient and HC (Supplemental Figure 5D). In the peripheral blood, the CDR3 lengths of IgM isotype in the patient were also shorter than in HC, though the difference did not reach statistical significance (Figure 6D). In accordance with the results of the BM, the patient peripheral IgM also had shorter *D_H_* segments than did HC (Figure 6E).

### The patient BM expressed high levels of IgG transcripts and contained abundant Ab-secreting cells.

**Figure 7:**
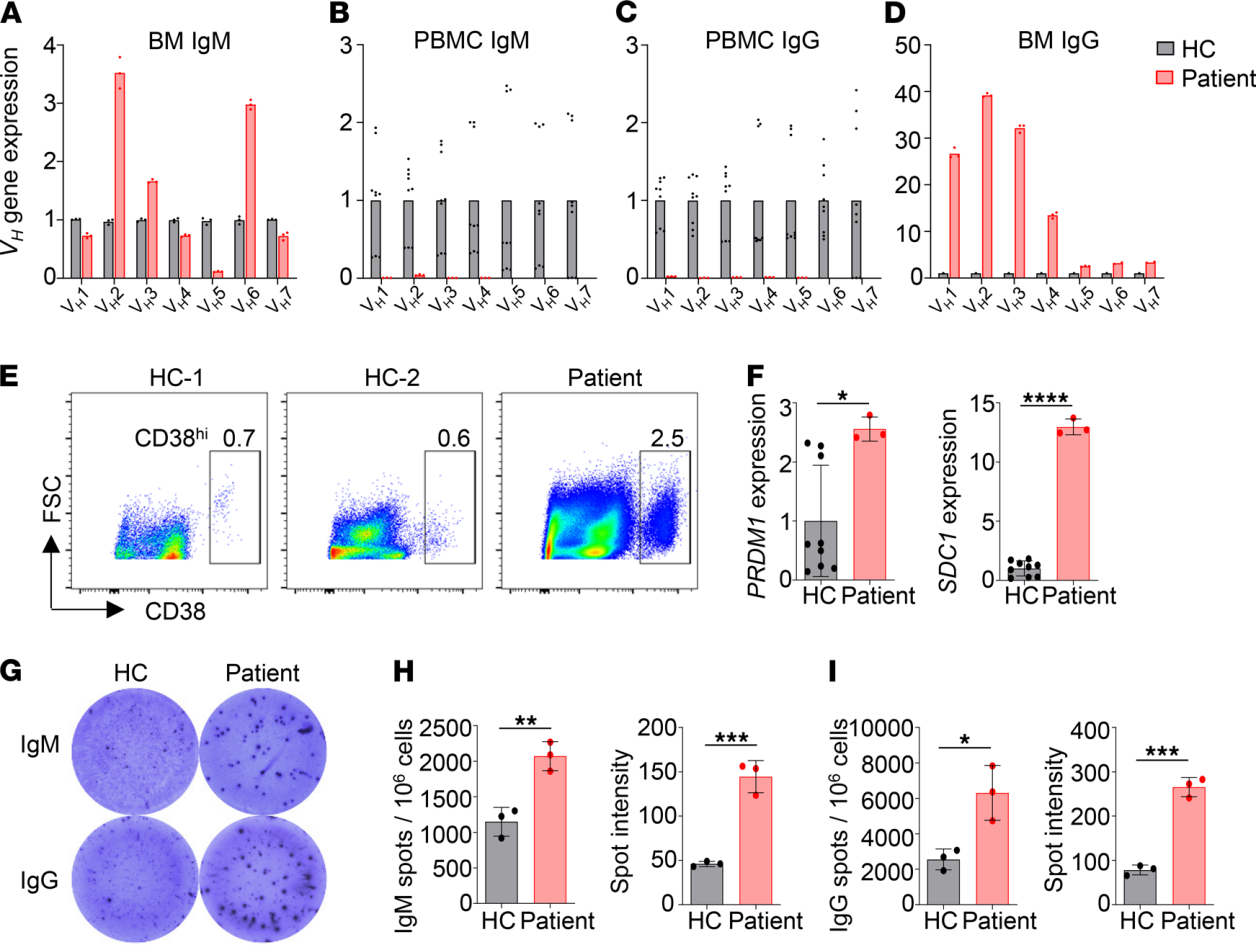
The patient BM expressed high levels of IgG transcripts and contained abundant Ab-secreting cells. Real-time PCR was performed to analyze transcript levels of individual *V_H_* genes of the BM IgM (**A**), peripheral IgM (**B**), peripheral IgG (**C**), and BM IgG (**D**) from an HC (**A**) or 3 HCs (**B**–**D**) and the patient. For comparison, transcript level of each *V_H_* gene in HCs was set to 1. *ACTIN* was used as an internal control. (**E**) FACS analysis of the CD38^+^ plasma cells in the BM of the patient and 2 HCs. (**F**) Real-time PCR analysis of *PRDM1* (BLIMP1) and *SDC1* expression in BM cells from 3 HCs and the patient. Mean ± SD is shown. (**G**) ELISPOT assay of IgM- and IgG-secreting plasma cells in the BM of an HC and the patient. (**H** and **I**) Mean ± SD of the numbers and sizes of the spots of triplicate wells. Statistical analyses were performed using Student’s *t* test. *P < 0.05; **P < 0.01; ***P < 0.001; ****P < 0.0001.

We next analyzed transcript levels of individual *V_H_* genes in the BM and peripheral blood using real-time PCR, after confirming the specificity of each of the *V_H_* gene primers (Supplemental Figure 6). As shown in Figure 7A, the patient BM expressed more *V_H_2* and *V_H_6* but fewer *V_H_5* transcripts of the IgM isotype as compared with HC. It should be noted that *V_H_* transcripts of the IgM isotype in the BM can be derived from both IgM^+^ cells and pre-B cells that express *VDJCμ* transcripts in the absence of light chain expression. Consistent with the greatly reduced number of B cells in the patient peripheral blood, the transcript levels of all *V_H_* genes, both for IgM (Figure 7B) and IgG isotypes (Figure 7C), were dramatically decreased in the patient compared with HC.

Remarkably, we found dramatically higher levels of *V_H_* transcripts, particularly *V_H_1*–*V_H_4*, of IgG isotype in the patient BM compared with HC (Figure 7D), suggesting that the patient BM contained many more IgG^+^ B cells than did HC. Since the levels of IgG *V_H_* transcripts in the patient peripheral blood were very low compared with HC (Figure 7C), the IgG^+^ cells in the patient BM were not recirculating B cells, but rather represented BM-resident cells. Further analysis revealed that the patient BM contained a much higher proportion of CD38^hi^ plasma cells compared with 2 HCs (Figure 7E) and expressed elevated levels of *PRDM1* (the gene coding BLIMP1) and *SDC1* (the gene coding CD138) transcripts (Figure 7F). An enzyme-linked immunospot assay (ELISPOT) confirmed the presence of increased number of plasma cells (Figure 7G) in the patient BM that secreted higher levels of IgM (Figure 7H) or IgG (Figure 7I) as compared with HC. These results demonstrate that despite the significantly reduced number of B cells in the patient peripheral blood, these B cells efficiently differentiated into plasma cells that resided in the BM and secreted large amounts of IgM and IgG.

### Increased frequency of DN B and memory B cells is strongly associated with decreased number and proportion of naive B cells in patients with PID.

**Figure 8:**
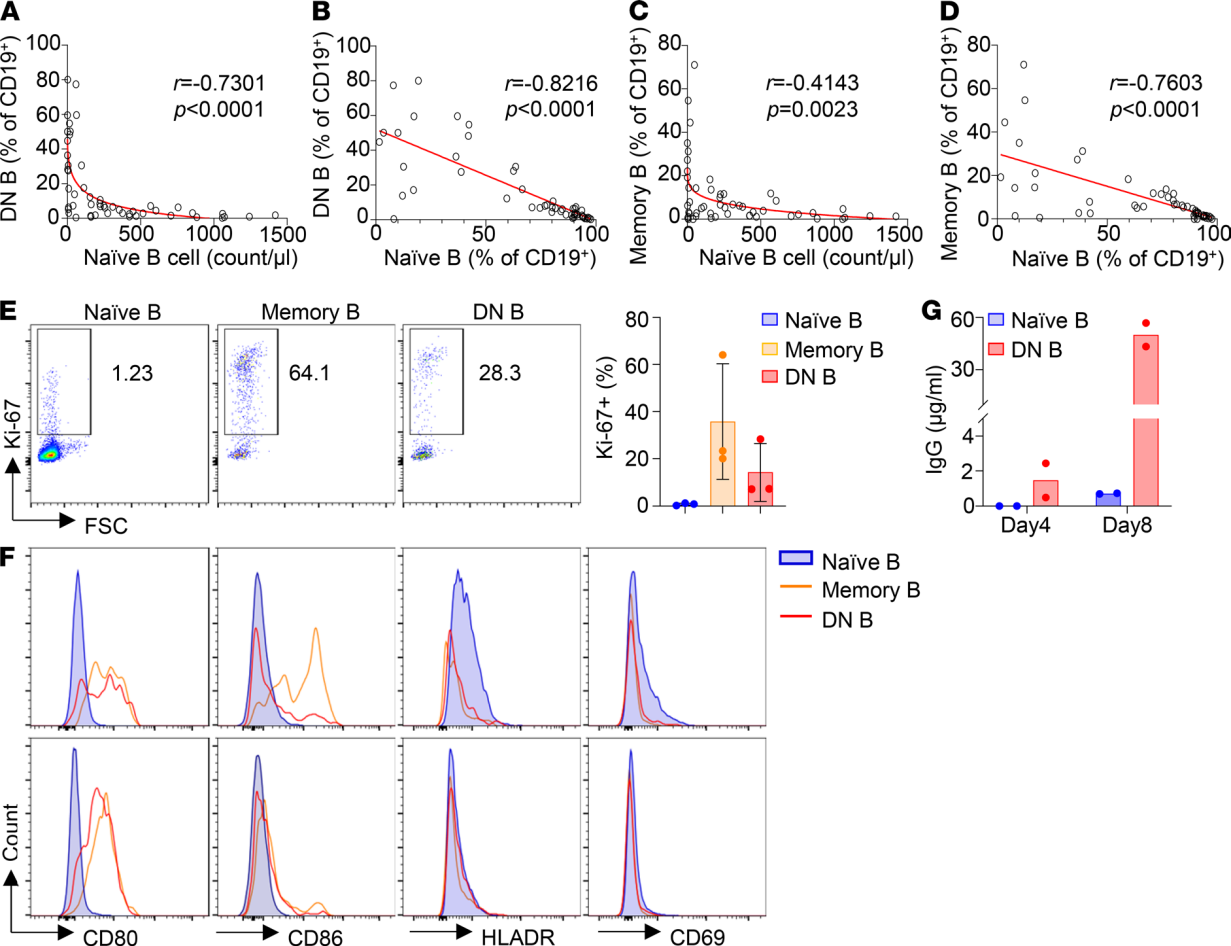
Increased frequency of DN B and memory B cells is strongly associated with decreased number and proportion of naive B cells in patients with PID. Comprehensive immune phenotype analyses were performed for 52 patients with PID (Supplemental Table 2). Spearman’s correlation analysis revealed a strong association of the increased proportion of DN B cells with decreased number (**A**) and proportion (**B**) of naive B cells. In addition, increased frequency of memory B cells was also associated with decreased number (**C**) and proportion (**D**) of naive B cells. Graphs (red lines) represent 4-parameter nonlinear regression (**A** and **C**) and linear regression (**B** and **D**), and Spearman’s rank correlation coefficient *r* and *P* values are shown. (**E**–**G**) Phenotypic and functional analysis of the DN B and memory B cells from 3 patients with PID. Compared with naive B cells, both memory B and DN B contained a higher proportion of Ki-67^+^ cells (**E**) and expressed elevated levels of the costimulatory molecules CD80 and CD86 (**F**). (**G**) Naive B and DN B cells were sorted and cultured in the presence of R848, BAFF, IL-21, IL-2, and IFN-γ and measured for IgG Abs in the culture supernatants.

The patient in the present study had a decreased proportion of naive B cells and an increased proportion of DN B cells in the peripheral blood (Figure 3, C and D) and produced elevated levels of Abs, including autoantibodies (Table 1 and Figure 7, G–I). A recent study has shown that the DN B cells found in the peripheral blood of patients with systemic lupus erythematosus (SLE) can efficiently differentiate into Ab-secreting cells upon in vitro stimulation (27). To explore whether the increased proportion of DN B cells is associated with changes in other B cell subsets, we performed detailed immune phenotype analysis of peripheral blood from 52 patients with PID (Supplemental Table 2). Remarkably, we found a strong association of the increased proportion of DN B cells with decreased number (Figure 8A) and proportion (Figure 8B) of naive B cells. Moreover, we also observed a correlation between elevated proportion of the memory B cells and reduced number and proportion of naive B cells (Figure 8, C and D). Notably, the proportion of both DN B and memory B cells increased sharply when the naive B cell counts were reduced below 200 cells/μL in the blood (Figure 8, A and C), indicating that the generation of DN B and memory B cells is highly dependent on B cell lymphopenic condition. In addition, the proportion of both DN B and memory B cells exhibited a rather simple inverse correlation with the proportion of naive B cells (Figure 8, B and D), suggesting that among the CD19^+^ B cells, a decrease of naive B is closely associated with an increase of DN B and memory B cells. These observations collectively suggest that B cell lymphopenic environment triggers the differentiation of naive B cells into DN B and memory B cells.

Compared with naive B cells, both DN B and memory B cells contained a much higher proportion of Ki-67^+^ cells (Figure 8E) and expressed elevated levels of the costimulatory molecules CD80 and CD86 (Figure 8F), indicating that these 2 subsets have an activated phenotype. We further sorted naive B and DN B cells and cultured these cells in the presence of TLR7 agonist and cytokines. We found that DN B cells secreted 1.5 μg/mL of IgG Abs by day 4 and 50 μg/mL by day 8 (Figure 8G). By contrast, naive B cells cultured under the same condition did not secrete any IgG Abs by day 4 and secreted only 0.7 μg/mL by day 8 (Figure 8G). These results demonstrate that the DN B cells can quickly differentiate into IgG-secreting plasma cells upon stimulation.

Among the 52 PID patients shown in Supplemental Table 2, 8 patients had a high proportion (>10%) of both DN B and memory B cells (indicated by *; patients with IVIG not counted). Five out of the 8 patients had higher levels of serum IgG and/or IgM compared with HC, and the other 3 patients had similar levels of IgG and/or IgM as HC. In addition, 4 of the 8 patients were tested for autoantibodies and 3 of them were positive. These observations are consistent with the hypothesis that elevated proportion of DN B and memory B cells is associated with increased production of Abs, including autoantibodies.

## Discussion

A number of missense and frameshift mutations in *RAG1* have been reported thus far to be associated with CID-G/AI (13, 28–33). To our knowledge, the present study is the first report of CID-G/AI caused by a homozygous splice site mutation. In contrast to the previously reported RAG1 deficiencies caused by missense mutations that affected its function, the patient of the present study had no mutations in the *RAG1* coding region and expressed normal RAG1 proteins but at significantly reduced levels. Based on the results of the sensitive EGFP reporter assay, we suggest that RAG1 protein levels in the patient BM cells were approximately one-thirtieth of that in HCs (Figure 2E). RAG1 protein level was likely reduced by the combined effects of NMD of the aberrantly spliced mRNA and decreased translational efficiency due to the presence of multiple ATGs with PTCs upstream of the authentic ATG. The residual RAG1 proteins allowed for low levels of V(D)J recombination, leading to CID-G/AI rather than SCID.

Similar to other CID patients with *RAG1* coding region mutations (34), the splice site mutation in *RAG1* caused a severe block of B cell development in the BM. Consequently, the total number of CD19^+^ B cells in the patient peripheral blood was reduced to less than 2% of the reference value by age 8. Analysis of IgM CDR3 in patient BM revealed significantly decreased clonal diversity for each of the *V_H_* families as compared with HCs. CDR3 clonality of the peripheral IgM was also significantly decreased in the patient relative to HC. Moreover, NGS of the patient peripheral *IgH* transcripts revealed that only 4% of the CDR3 were unique, in contrast to 61.2% and more than 80% being unique in previously reported HCs (34, 35). The high proportion of the B cells with identical CDR3 sequences in the patient peripheral blood suggests that these B cells had undergone clonal expansion, possibly through homeostatic proliferation. Because of the severe B cell lymphopenia, the reduced Ig repertoire in the patient blood could also be due to a much lower number of templates that could be amplified.

The number of T cells in the periphery was also reduced to less than 2% of the reference value. A substantial proportion of patient peripheral T cells had a CD4 and CD8 DN phenotype, and almost all of them expressed a γ/δ TCR. Recently, mice with hypomorphic *RAG1* mutations were also shown to have an increased proportion of DN γ/δ T cells in the periphery (23). Therefore, both in humans and in mice, γ/δ T cells appear to be less affected than α/β T cells by the reduced RAG1 activity. One possibility is that during α/β T cell development, TCRβ undergoes rearrangement first, followed by pre-TCR selection and then TCRα rearrangement, whereas during γ/δ T cell development, TCRδ and TCRγ loci undergo rearrangements simultaneously and thus only require RAG expression for a short period (36). Expansion of γ/δ T cells has been shown to be associated with many autoimmune diseases (37, 38), and their increase in the patient in the present study, who manifested with autoimmune symptoms, is consistent with these earlier observations.

The patient BM and peripheral *IgH* genes had shorter CDR3 likely due to increased exonuclease nibbling of *D_H_* segments. It has been shown that the RAG1-RAG2 complex remains associated with the DNA ends after introducing DNA DSBs, which functions to protect the DNA from exonuclease-mediated digestion (5, 6). Previous studies have shown that some of the RAG mutants fail to remain anchored to the DNA DSBs, resulting in shorter CDR3 sequences (39, 40). However, the likely unique nature of the splice site mutation described in the current study allowed the expression of WT RAG1 but at greatly reduced levels. Since RAG1-RAG2 binding affinity is quite high (*K_D_* ~0.4 μM) (41), one possibility for the shorter CDR3 in this patient is that the presence of excessive free RAG2 might affect the stability of the RAG1-RAG2 complex if the free RAG2 attempts to bind to the RAG1 within the complex.

Despite B cell lymphopenia in the periphery, the patient produced elevated levels of IgM and IgG Abs and autoantibodies. We found that the patient BM contained abundant IgG^+^ cells and Ab-secreting plasma cells, indicating that the few B cells in the patient periphery efficiently differentiated into plasma cells. One striking feature of the patient peripheral B cells is that more than 50% of them are CD27^–^IgD^–^ DN B cells. Such DN B cells have previously been found to be increased in patients with autoimmune/autoinflammatory diseases, such as SLE (42), mixed connective tissue disease (43), and multiple sclerosis (44), and in elderly people (22, 45). The latter also have an inflammatory microenvironment, with elevated levels of IL-6, C-reactive protein, and TNF-α in the blood (45, 46). Therefore, the increase of DN B cells appears to be associated with inflammation. A more recent study demonstrated that the DN B cells in patients with SLE were precursors of plasma cells (27). These studies also revealed that the DN B cells in the patients with SLE exhibited a DN2 phenotype (CXCR5^–^CD21^–^CD19^hi^). Upon stimulation with TLR7, IL-21, and IL-10, these DN2 cells efficiently differentiated into plasma cells that secreted large numbers of Abs, including autoantibodies. Notably, the DN B cells of our patient also expressed high levels of CD19 and may thus resemble the DN2 cells found in the patients with SLE. Consistently, we demonstrated that the DN B cells sorted from the peripheral blood of 2 patients with PID rapidly differentiated into IgG-secreting plasma cells upon stimulation (Figure 8G). Therefore, the abundant plasma cells detected in the patient BM could be derived from the DN2-like cells present in the periphery.

CID-G/AI and AS patients with RAG1 deficiency commonly present with autoimmune manifestations, and 26.3% of them show hypergammaglobulinemia despite profound B lymphocytopenia (19, 47). Some features of autoreactive *IgH* CDR3, including increased CDR3 length and frequent usage of *V_H_*4-34, *V_H_*3-9, *V_H_*4-31, and *V_H_*3-23, have been observed in a fraction of CID-G/AI patients (34, 35). The patient in the present study produced autoantibodies, including ANA and pANCA. However, the CDR3 length was not increased but rather decreased in the patient, and the frequency of *V_H_*4-34 usage for IgG isotype showed no difference between the patient and HC. While certain *V* genes may exhibit autoreactivity, it is likely that autoreactivity can also be generated by other *V* genes because of the random nature of V(D)J recombination, the introduction of junctional sequences, the different light chains used, and the somatic hypermutation. Under normal conditions, however, autoreactive B cells are eliminated by clonal deletion, receptor editing, or functional inactivation (anergy) (48). Receptor editing is a process where the *V* gene of an already rearranged light chain is replaced by an upstream *V* gene, thus creating a BCR with a new, potentially no longer autoreactive, specificity (48). This process is mediated by RAG1 and RAG2, and indeed mice with hypomorphic *RAG1* mutations have been shown to be defective in receptor editing (23, 49). It is likely that RAG-deficient humans also have defects in receptor editing important for the removal of autoreactive B cells.

**Figure 9:**
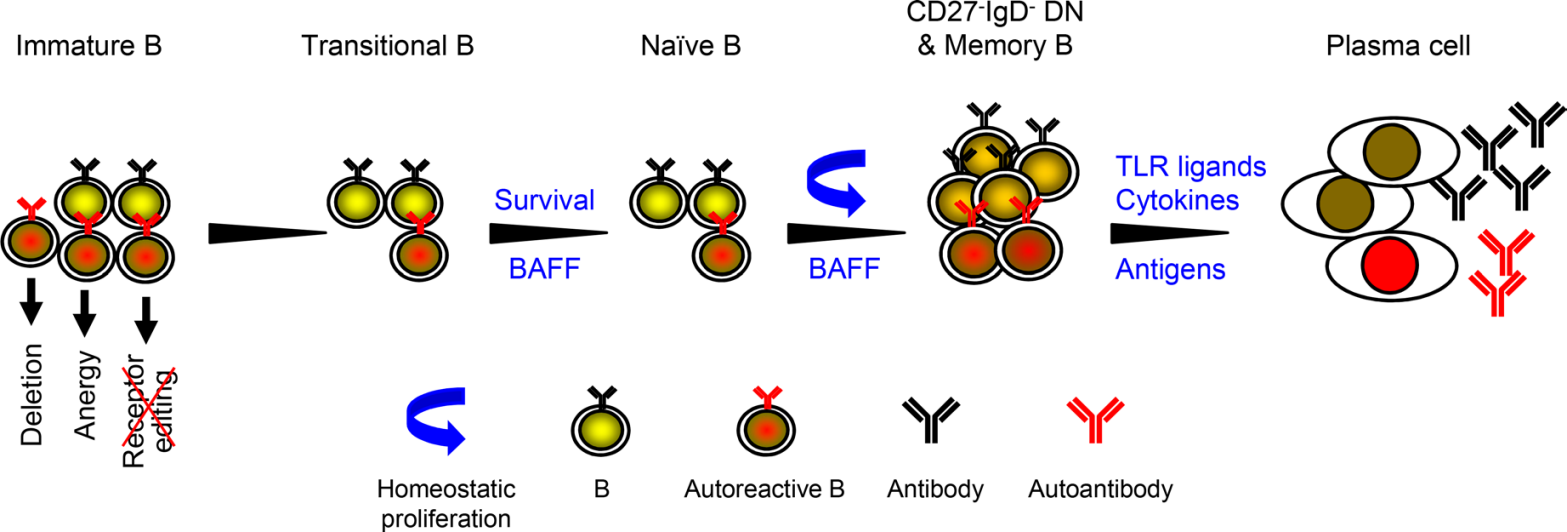
A model for autoantibody production in patients with hypomorphic *RAG* mutations. Autoreactive immature B cells are normally eliminated by at least 3 mechanisms: deletion (apoptosis), anergy (functional inactivation), and receptor editing (48). Receptor editing is mediated by RAG1 and RAG2 and functions to remove autoreactive immature B cells by changing the specificity of the BCR. As proposed by Notarangelo et al. (4) and others (23, 49), hypomorphic *RAG* mutation impairs receptor editing and thus allows some autoreactive immature B cells to exit the BM and become TrB cells. Due to the B cell lymphopenic environment that causes increased levels of BAFF, autoreactive TrB cells were able to survive and become mature naive B cells. We think that these naive B cells possibly acquire the CD27^–^IgD^–^ DN or memory B phenotype as a result of homeostatic proliferation. Such DN B and memory B cells can efficiently differentiate into plasma cells upon stimulation with TLR ligands and cytokines (Figure 8G) or with foreign or self-antigens, and secrete large numbers of Abs, including autoantibodies.

As proposed in earlier studies, *RAG* mutation impairs receptor editing (23, 49) and thus allows some autoreactive immature B cells to exit the BM and become transitional B (TrB) cells. Such autoreactive TrB cells normally express low levels of BAFF receptor and thus have a disadvantage in BAFF-induced survival compared with non-autoreactive TrB cells (4, 50, 51). In the B cell lymphopenic environment, however, serum BAFF levels are elevated (52) (Supplemental Figure 3), allowing autoreactive TrB cells to survive and become mature naive B cells (53, 54). These naive B cells undergo homeostatic proliferation because of the lymphopenic environment and the presence of increased levels of BAFF (55–58). Based on these earlier observations in mice and humans and the findings of the present study that the increased proportion of DN B and memory B cells correlated with the decrease of naive B cells, we propose that the homeostatic proliferation of naive B cells results in the generation of DN B and memory B cells (Figure 9). Upon stimulation with TLR ligands and cytokines (27) (Figure 8G) or with self- or foreign antigens, these DN B and memory B cells can efficiently differentiate into plasma cells and secrete large numbers of Abs, including autoantibodies (Figure 9).

In summary, the present study has identified the first case to our knowledge of CID-G/AI caused by a novel splice site mutation in the intronic region of *RAG1* without any mutations in the *RAG1*-coding region. This splicing mutation likely caused reduced RAG1 expression and impaired the V(D)J recombination processes, resulting in developmental block of B and T cells, reduced *IgH* repertoire diversity, and altered peripheral blood B and T cell compartments. Although the peripheral B cell numbers were markedly reduced, these B cells acquired a DN phenotype and could efficiently differentiate into IgG-secreting plasma cells upon stimulation. Our findings provide important new insights into the mechanisms of elevated Ab production and autoimmunity in patients with lymphopenic PID.

## Methods

### Routine evaluation of immunological function.

A complete blood count was evaluated using a cell counter with anticoagulated whole blood. Serum IgG, IgA, and IgM levels were determined by an automated clinical chemistry analyzer (Erba, model XL-200) as previously described (59). Serum IgE was measured by UniCAP (Pharmacia). ANA, anti-mitochondrial Ab, anti-nucleosome Ab, cytoplasmic anti-neutrophil cytoplasmic Ab, pANCA, PR3-proteinase, and serum autoantibodies against dsDNA, ribonucleoprotein (RNP), extractable nuclear antigen (RNP/Sm, Sm, SSA, SSB, Scl-70, Jo-1), glomerular basement membrane, proliferating cell nuclear antigen, and myeloperoxidase were detected using immunoblot and ELISA kits (EUROIMMUN) according to the manufacturer’s instructions. The direct Coombs test was performed using a commercial kit (Brother Biotech).

### Genetic analysis.

WES and genetic analysis were performed as previously described (59). Briefly, genomic DNA was extracted from the whole blood of the patient and his parents, enriched for the target region of the consensus coding sequence exons, and then sequenced with the HiSeq 2000 sequencer (Illumina). The raw data were mapped to the human reference genome sequence. Data analyses were performed by a bioinformatics team in our clinical genetics laboratory. Nucleotide changes observed in more than 5% of aligned reads were called and reviewed by using NextGENe software (SoftGenetics).

### Analysis of RAG1 mRNA splicing and expression.

Total RNA was extracted from BMMNCs of the patient and an age-matched HC and from Nalm6 cells (a human pre-B cell line) using RNAiso Plus (TaKaRa Bio) and digested with DNase I (TIANGEN Biotech) to remove contaminating genomic DNA. The total RNA was then reverse-transcribed using PrimeScript 1st strand cDNA Synthesis Kit (TaKaRa Bio) and amplified with a forward primer located within exon 1 (5′-GGAGAGAGCAGAGAACACACT-3′) and a reverse primer located within exon 2 (5′-CAAAGGATCTCACCCGGAACA-3′). PCR was performed using LA-Taq (TaKaRa Bio) under the following conditions: 95°C for 2 minutes followed by 98°C for 10 seconds and 68°C for 5 minutes, 30 seconds, for 32 cycles. The PCR products were then subcloned into pMD20-T vector (TaKaRa Bio) and sequenced to detect aberrantly spliced transcripts.

### Construction of the RAG1-EGFP reporter vectors.

Amplification of the *EGFP* gene and *RAG1* genomic fragment was performed with the high-fidelity KOD-plus polymerase (TOYOBO) to minimize PCR errors. *EGFP* was amplified using 5′-GCGTCGACGAGAGCGACGAGAGCGGC-3′ (including a SalI site) and 5′-CGGGATCCTTAGCGAGATCCGGTGGAG-3′ (including a stop codon and a BamHI site) primers from pCDH-EGFP vector under the following conditions: 95°C for 2 minutes followed by 94°C for 30 seconds and 68°C for 1 minute for 32 cycles. The PCR product was subcloned into pMD20-T vector for sequence verification, then inserted into pFlag-CMV-5a (MilliporeSigma) to generate the pFlag-CMV-5a-EGFP expression vector. A human *RAG1* genomic fragment containing exon 1, intron 1, and part of exon 2 (including the translation initiation site) was amplified using 5′-ATTTGCGGCCGCAGAGGGCAAGGAGAGAGCAG-3′ (including a NotI site) and 5′-GCGTCGACCATGCTGGCTGAGGTACCTG-3′ (including the translation initiation site ATG and a SalI site) primers and genomic DNA extracted from BMMNCs of the patient and HC. The PCR was carried out at 95°C for 2 minutes followed by 94°C for 30 seconds and 68°C for 7 minutes for 32 cycles. The amplified human *RAG1* genomic fragment was subcloned into a pMD20-T vector for sequence verification and then inserted upstream of and in frame with the *EGFP* gene in pFlag-CMV-5a-EGFP to generate the pFlag-CMV-5a-RAG1-EGFP reporter vector.

### Transfection of the RAG1-EGFP reporter vector and detection of EGFP expression.

293T cells in 6-well plates were transfected with WT or mutant pFlag-CMV-5a-RAG1-EGFP plasmid (2 μg per well) using a Lipofectamine 2000 transfection kit (Thermo Fisher Scientific) according to the manufacturer’s protocol. EGFP reporter expression was analyzed by fluorescence microscopy (Nikon) and flow cytometry (BD Biosciences) at different time points (24 hours, 48 hours, and 72 hours) after the transfection.

### Abs and flow cytometry.

Single-cell suspensions from the BM and peripheral blood were first incubated with human BD Fc block (BD Biosciences) to block Fcγ receptor and then stained with the following anti-human Abs: CD19-APC (clone HIB19, BioLegend), CD34-PE (clone 563, BD Biosciences), CD20-FITC (clone 2H7, BioLegend), CD27-APC/Cyanine7 (clone O323, BioLegend), IgD-PE (clone IA6-2, BD Biosciences), IgM-FITC (clone G20-127, BD Biosciences), CD43-PE (clone CD43-10G7, BioLegend), CD38-PE/Cy7 (clone HIT2, BioLegend), CD38-Percp/Cy5.5 (clone HIT2, BD Biosciences), CD24-PE (clone ML5, BD Biosciences), CD27-V450 (clone M-T271, BD Biosciences), IgD-BV510 (clone IA6-2, BD Biosciences), IgG-PE/Cy7 (clone G18-145, BD Biosciences), Ki-67–PE (clone Ki-67, BioLegend), CD80-FITC (clone L307.4, BD Biosciences), CD86-PE (clone IT2.2, BD Biosciences), HLADR-PE (clone G46-6, BD Biosciences) and CD69-PE/Cy7 (clone FN50, BD Biosciences). 7-AAD Viability Staining Solution (eBioscience, Thermo Fisher Scientific) was used for live versus dead cell discrimination. Samples were analyzed with a FACSVerse flow cytometer (BD Biosciences) using the FACSuite software. Data analysis was performed with FlowJo 10 software (Treestar).

### Analysis of BCR repertoire diversity and V_H_ gene expression.

To analyze the BCR repertoire, we performed *IgH* CDR3 spectratyping as described (26, 60). Briefly, cDNAs of PBMCs and BMMNCs served as templates for PCRs with the use of forward primers specific for each of the 7 *V_H_* gene families and fluorescent reverse primers for the IgM and IgG isotypes (26). The amplified products were analyzed by capillary electrophoresis and GeneMapper software (Applied Biosystems, Thermo Fisher Scientific). For sequencing, cDNAs of BMMNCs and PBMCs were amplified with the same forward primers and common reverse primers specific for the IgM and IgG isotypes. The PCR products were then subcloned into pMD20-T vectors and sequenced. Sequences were compared with the IMGT database, and the productive sequences were used to analyze CDR3 lengths. For quantitative analysis of individual *V_H_* gene transcripts, we performed real-time PCR using *V_H_* forward primers and reverse primers for the IgM and IgG isotypes with *ACTIN* as an internal control.

### ELISPOT assay.

This assay was performed as described previously (61). Briefly, multiscreen high-throughput screening plates (MilliporeSigma) were coated with 50 μg/mL of goat anti-human Ig (Southern Biotech). Serially diluted BMMNCs were added to individual wells in triplicate and then incubated at 37°C for 2 hours in a CO_2_ incubator. The plates were further incubated with biotin-conjugated anti–human IgM or IgG (Southern Biotech), followed by alkaline phosphatase–conjugated streptavidin (Southern Biotech). Spots were revealed by 5-bromo-4-chloro-3-indolyl phosphate/nitro blue tetrazolium reagent (MOSS Inc.), and colonies were counted by using an ELISPOT reader (AID).

### Cell culture.

293T cells were obtained from American Type Culture Collection (CRL-3216) and cultured in DMEM supplemented with 10% heat-inactivated fetal bovine serum (Gibco, Thermo Fisher Scientific). Nalm6 human pre-B cells (RCB1933, RIKEN Bioresource Research Center) were cultured in RPMI 1640 (Gibco, Thermo Fisher Scientific) supplemented with 10% heat-inactivated fetal bovine serum, antibiotics (100 U/mL penicillin and 100 μg/mL streptomycin), and 5 × 10^−5^ M 2-mercaptoethanol. FACS-sorted naive B and DN B cells were cultured as Nalm6 and supplied with TLR7 agonist R848 (1 μg/mL, Invivogen), BAFF (10 ng/mL, R&D Systems, Bio-Techne), IL-21 (10 ng/mL, R&D Systems, Bio-Techne), IL-2 (50 ng/mL, R&D Systems, Bio-Techne), IFN-γ (20 ng/mL, Peprotech), and goat F(ab′)_2_ anti-human IgM (10 μg/mL, SouthernBiotech). Four days later, the supernatants were collected, and the cells were further cultured in fresh media containing R848 and cytokines but without F(ab′)_2_ anti-IgM. After 4 additional days, the culture supernatants were collected, and the quantity of IgG Abs was analyzed by ELISA.

### ELISA.

Briefly, 96-well ELISA plates (Costar, Corning) were coated with goat anti–human Ig (SouthernBiotech) in PBS at 4°C overnight, then blocked with 1% BSA blocking buffer at room temperature for 1 hour. The culture supernatants and serially diluted standards were then added and incubated at room temperature for 1 hour. After washing, biotin-conjugated anti–human IgG (SouthernBiotech) Ab was added and incubated at room temperature for 1 hour. After washing, avidin-HRP (BioLegend) was added to the plates, incubated for 30 minutes, and visualized with TMB substrate reagents (Invitrogen, Thermo Fisher Scientific). OD_450_–OD_570_ was measured by a spectrophotometer (Bio-Rad). The amount of Ig in duplicate wells was calculated based on the standard.

### NGS of the patient peripheral and BM IgH transcripts.

For NGS of *IgH* transcripts, an equal amount of total RNA extracted from the patient BM and peripheral blood was used as template to perform dimer-avoided-multiplex PCR according to the manufacturer’s protocol (iRepertoire Inc.). PCR products were purified and sequenced (Illumina NovaSeq 6000). Raw sequences were filtered for PCR errors. Graphical representation of tree maps was generated using either the iRepertoire software or Microsoft Excel. The diversity index was defined as 100 – area under the curve between the percentage of total and unique CDR3 sequences. D50 represents the number of the most dominant unique CDR3s among all unique CDR3s that make up 50% of the total effective reads. High diversity index and D50 are associated with more diversity. The Gene Expression Omnibus accession number of the NGS data of the *IgH* repertoire in the patient peripheral blood and BM is GSE181689.

### Statistics.

Data were analyzed with GraphPad Prism 6. Results are presented as mean ± SD. Statistical analysis of the data was performed with 2-tailed Student’s *t* test. Correlation between naive B and DN B or memory B cells was determined with 4-parameter nonlinear or linear regression and 2-tailed Spearman’s correlation analysis. *P < 0.05; **P < 0.01; ***P < 0.001; ****P < 0.0001. *P* values less than 0.05 are considered statistically significant.

### Study approval.

All experiments were approved by the Ethics Committee of Fudan University and the Ethics Committee of the Children’s Hospital of Fudan University (registration no. NCT03383380). Informed consent was obtained from the parents of the patients and of the age-matched control without immunological diseases. Written informed consent was obtained from the parents of the patient for use of the photograph of the patient.

## Author contributions

QM and JYW designed the study; QM and XM performed experiments; YL, NL, EX, and SY analyzed the data; QZ, YW, WW, MY, HZ, and JS provided clinical data and patient samples; QZ and XW supervised the research; QM wrote the manuscript; and JYW edited and finalized the manuscript.

## Supplementary Material

Supplemental data

## Figures and Tables

**Table 1:**
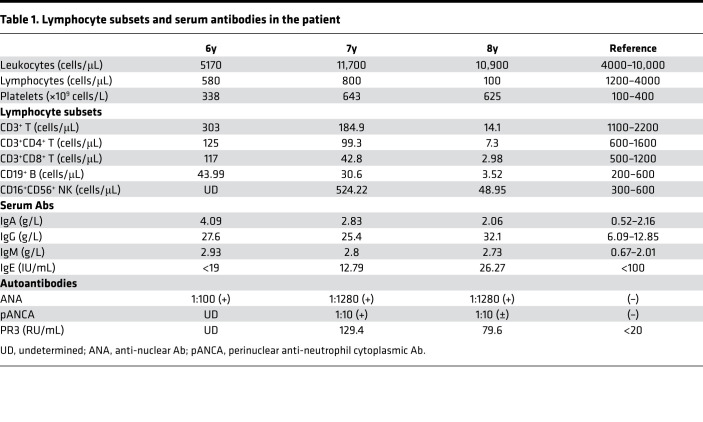
Lymphocyte subsets and serum antibodies in the patient

**Table 2:**
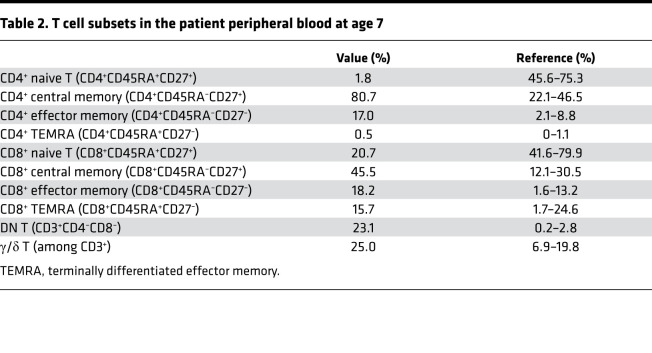
T cell subsets in the patient peripheral blood at age 7

**Table 3:**
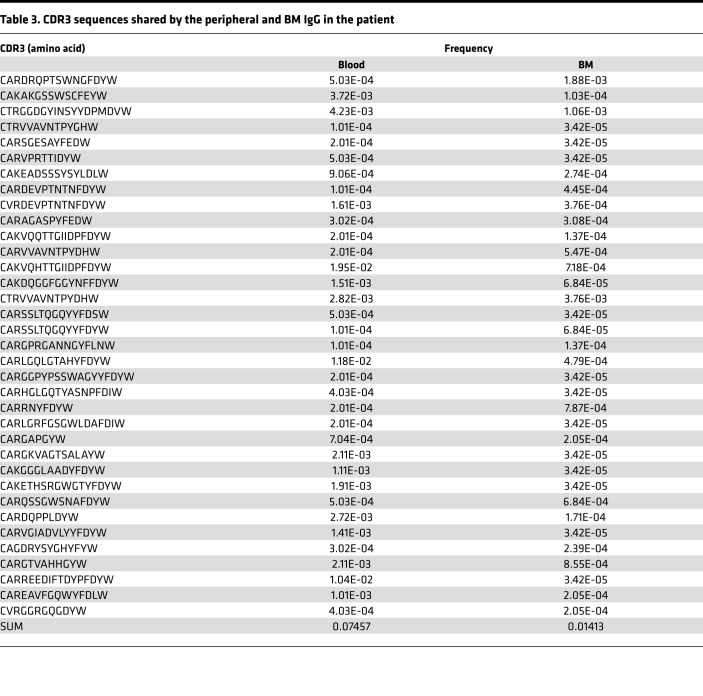
CDR3 sequences shared by the peripheral and BM IgG in the patient

## Version 1 10/08/2021

Electronic publication
