# Supplementary material for: RAG1 splicing mutation causes enhanced B cell differentiation and autoantibody production
Source: JCI Insight. 2021 Oct 8;6(19):e148887. doi: 10.1172/jci.insight.148887 (PMC8525647; doi:10.1172/jci.insight.148887)
Supplement: Supplemental data [file jciinsight-6-148887-s028.pdf]

Exon 1

GAAACAAGAGGGCAAGGAGAGCAGAGAACACACTTTGCCTTCTCTTTGGTA
TTGAGTAATATCAACCAAATTGCAGACATCTCAACACTTTGGCCAGGCAGCCTG
CTGAGCAAGG**G**TACACTCATACTTTTCATGCCTCGAGCCGAAATATTTATTACAT
TTTTATGTTTCTAACTAGAAGTGCTTGAGCTTTTTTCCTTCCAGGTGATGAGGG
GATGGAATGAGCAAAGCTACATCAATTTTTTTTAATGTATGAAAATAAAAAAGG
TACAAGAGGCCAAGTTTAGGGCCACTGAAGGTTCATAGAAAGATGCAAAATATC
TGAATTACTATAAATGAATGCTATTGTCAGAGGAAAGGTTTAAGGAGTGCTTCTT
GAATGAATGTGTACAAATCAGCAGAAGGTACCTCAGCCAGC**ATG**GCAGCCTCT
TTCCCACCCACCTTGGGACTCAGTTCTGCCCCAGATGAAATTCAGCACCCACAT
ATTAAATTTTCAGAATGGAAATTTAAGCTGTTCCGGGTGA

Exon 2

Supplemental Figure 1 Product of the aberrantly spliced *RAG1* mRNA in the patient. The sequence includes the entire exon 1 (highlighted in grey), retained intron 1 (black) and part of exon 2 (highlighted in grey). The mutated nucleotide is shown in red. Eleven out-of-frame ATGs in intron 1 are underlined in red and an ATG in intron 1 that is in-frame with the authentic ATG (highlighted in blue) is underlined in black. Eleven premature stop codons are squared.

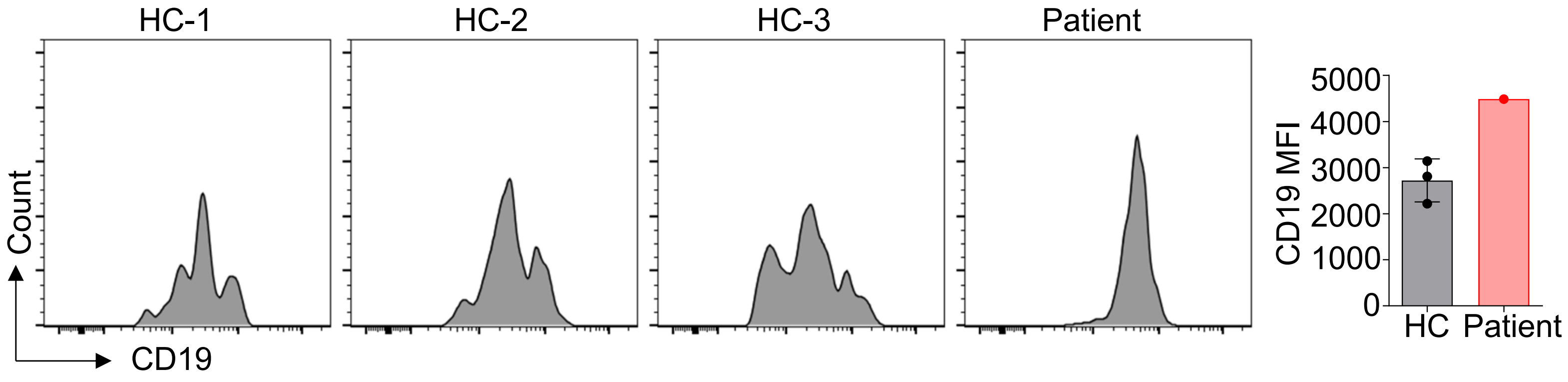

Supplemental Figure 2 The patient IgD$^-$CD27$^-$ DN B cells expressed higher levels of CD19 than did those from HC. The DN B cells from HC and the patient (Fig. 3B, lower panels) were analyzed for their CD19 expression.

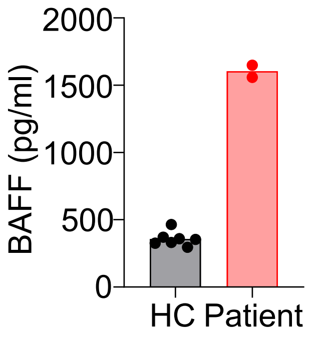

Supplemental Figure 3 The patient serum contained an elevated level of BAFF than the sera from 7 HC. The concentration of BAFF was analyzed using ELISA. The patient sample was independently analyzed twice.

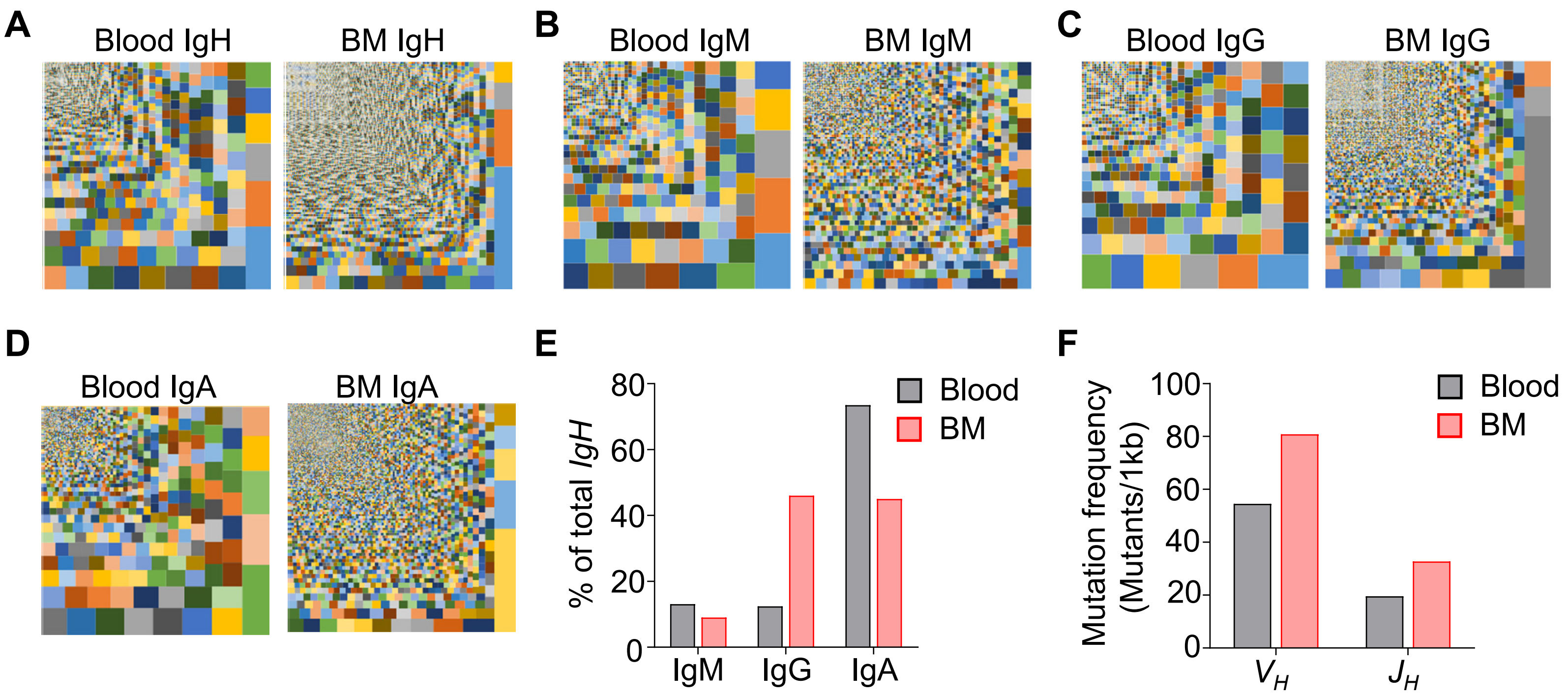

Supplemental Figure 4 (**A-D**) Tree maps showing CDR3 amino acid sequences from the patient peripheral blood and BM. (**A**) Total IgH, (**B**) IgM, (**C**) IgG and (**D**) IgA. Each rectangle represents a unique CDR3 amino acid sequence and the size of the rectangle represents the relative frequency of an individual CDR3. (**E**) Frequency of IgM, IgG and IgA sequences among all CDR3s in the patient peripheral blood and BM. (**F**) Mutation frequency in the V_H and J_H genes of the patient peripheral blood and BM.

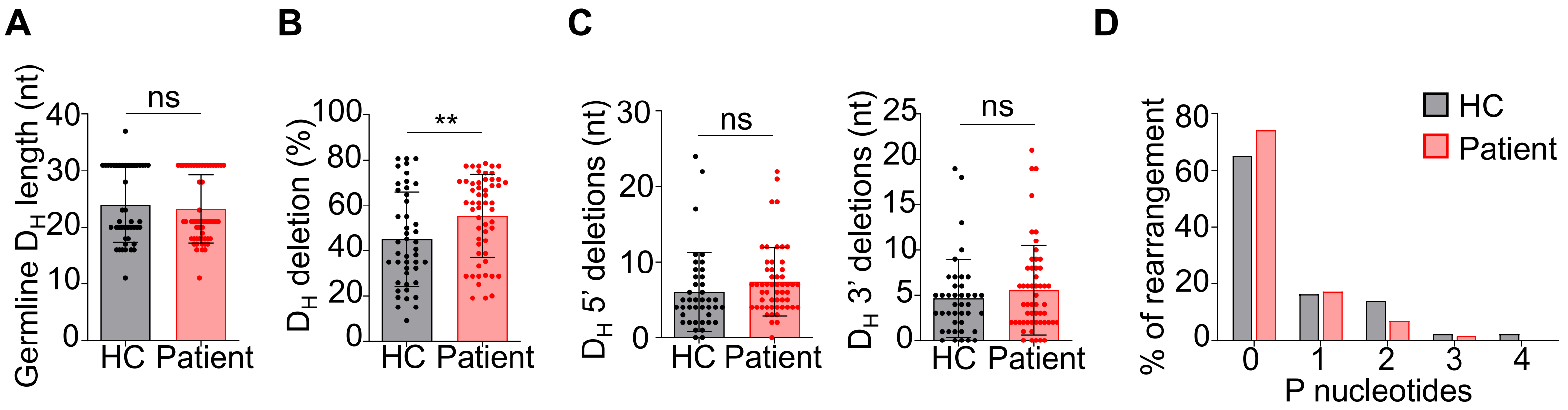

Supplemental Figure 5 *IgH* CDR3 profiles in the BM. (**A**) Average length of the germline D_H segments used by the patient and HC. (**B**) Proportion of the deleted nucleotides (nt) from germline D_H segment. (**C**) Number of nt deleted from the 5' (left) and 3' (right) ends of the D_H segments in the patient and HC. (**D**) The distribution of P-nucleotide addition. Mean ± SD is shown. $^{**}p < 0.01$; ns, not significant.

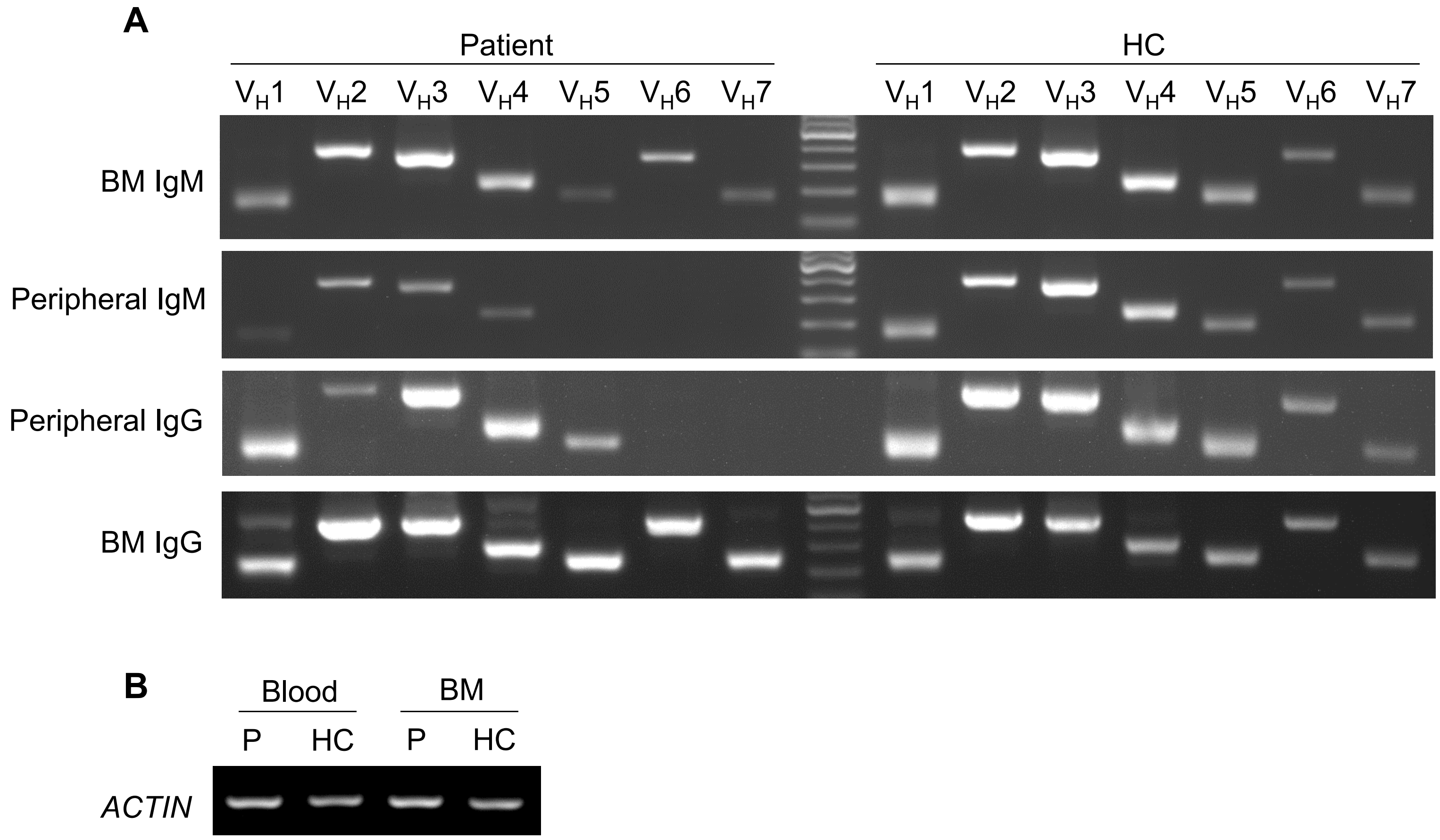

Supplemental Figure 6 Amplification of V_H genes using cDNA derived from the BM and peripheral blood of the patient and HC. (**A**) V_H-specific forward primers and a Cμ (for IgM) or a Cγ (for IgG) reverse primer were used for amplification. (**B**) As a control, the *ACTIN* gene was amplified from the same cDNA.

Supplemental Table 1. Serum Ig levels in the patient at age 7

IgG subclass	Value (g/L)	Reference (g/L)
IgG_1	20.5	3.77-11.31
IgG_2	1.02	0.68-3.88
IgG_3	0.945	0.158-0.890
IgG_4	0.0687	0.012-1.699

Supplemental Table 2. Clinical and immunological features of the PID cohort

	Sex	Age	Genetic defects	Clinical features	B cell (cells/μl)	Reference values	T cell (cells/μl)	Reference values	Naive B (% of $CD19^+$)	Naive B (cells/μl)	DN B (% of $CD19^+$)	DN B (cells/μl)	Memory B (% of $CD19^+$)	Memory B (cells/μl)	IgG (g/L)	Reference values	IgM (g/L)	Reference values	IgA (g/L)	Reference values	Auto-antibody
PID001*	M	8Y	RAG1	Fever; infections; granulomas	4	300-600	14	1100-2200	17.3	0.60896	59.5	2.38	22.1	0.884	32.1	6.09-12.85	2.73	0.67-2.01	2.06	0.52-2.16	ANA, pANCA, Coombs
PID002	M	9M	RAG1	Recurrent infections	801	300-800	1958	1800-3300	70.9	567.909	7.1	56.871	11.8	94.518	14.66	3.50-8.90	3.38	0.36-1.20	1.12	0.06-0.54	Coombs
PID003	M	11Y	PIK3R1	CVID	32	300-600	796	1100-2200	42.3	13.71789	54.8	17.536	2.54	0.8128	0.54	6.09-12.85	0.1	0.67-2.01	0.14	0.52-2.16	SSA
PID004*	F	1Y5M	Unknown	Recurrent infections	397	300-600	1984	1900-2900	12.1	48.037	13.9	55.183	71	281.87	38.5	5.52-11.46	2.98	0.60-2.12	1.65	0.06-0.74	ANA
PID005*	F	4Y4M	Unknown	Eosinophilia	739	300-600	1812	1900-2900	7.97	58.8983	77.4	571.986	14.3	105.677	24.35	4.95-12.74	1.08	0.65-2.01	1.65	0.33-1.89	SSB
PID006	M	4Y9M	PIK3CD	Recurrent fever and diarrhea	211	300-600	1303	1500-2900	76.8	162.048	8.01	16.9011	13.6	28.696	19[#]	4.95-12.74	3.85	0.65-2.01	1.54	0.33-1.89	PR3, GBM
PID007	M	13Y	Unknown	Hypogammaglobulinemia	303	300-600	1282	1100-2200	84.7	256.641	6.12	18.5436	5.32	16.1196	5[#]	6.98-14.26	1.54	0.56-2.16	2.09	0.92-2.50	(-)
PID008	F	13Y	TNFRF13B	Thrombocytopenic	301	300-500	680	1300-2200	74.6	224.546	6.86	20.6486	11.4	34.314	10.79[#]	6.98-14.26	0.54	0.56-2.16	1.32	0.92-2.50	(-)
PID009	M	16Y	Unknown	CVID	203	300-600	759	1100-2200	93.9	190.617	1.78	3.6134	1.06	2.1518	ND	7.54-16.62	0.02	0.72-2.28	0.05	0.89-3.24	(-)
PID010	M	5Y6M	Unknown	Recurrent fever; encephalopathy	494	300-600	2112	1500-2900	90.6	447.564	3.95	19.513	3.21	15.8574	9.68[#]	4.95-12.74	0.82	0.65-2.01	1.65	0.33-1.89	(-)
PID011	M	15Y	PIK3CD	Hyper IgM syndrome	169	300-600	909	1100-2200	66.5	112.385	17.5	29.575	5.69	9.6161	5.26[#]	7.54-16.62	18.3	0.72-2.28	0.99	0.89-3.24	(-)
PID012	F	9M	Unknown	Thrombocytopenic; agranulocytosis	1307	300-800	3371	2800-3500	95.1	1242.957	0.92	12.0244	1.44	18.8208	19.37	3.50-8.90	0.46	0.36-1.20	0.11	0.06-0.54	(-)
PID013	F	4Y8M	PIK3CD	Hypogammaglobulinemia; lymphadenopathy	36	300-600	746	1900-2900	42	15.12	48.3	17.388	7.91	2.8476	3.04[#]	4.95-12.74	11.06	0.65-2.01	0.24	0.33-1.89	(-)
PID014	M	4Y4M	PIK3CD	APDS	278	300-600	1138	1500-2900	80.5	223.79	10.6	29.468	6.66	18.5148	9.15[#]	4.95-12.74	3.82	0.65-2.01	0.55	0.33-1.89	(-)
PID015	M	2Y	Unknown	Recurrent fever and diarrhea	310	300-600	2696	1500-2900	80.2	248.62	7.23	22.413	11.6	35.96	19.9[#]	5.52-11.46	1.25	0.60-2.12	2.97	0.06-0.74	(-)
PID016	M	5Y4M	Unknown	Recurrent fever	285	300-600	901	1500-2900	77.7	221.445	8.46	24.111	10	28.5	9.68	4.95-12.74	0.96	0.65-2.01	2.31	0.33-1.89	(-)
PID017	M	3M	WAS	WAS	301	300-800	2143	1800-3300	91.3	274.813	2.95	8.8795	2.43	7.3143	21.58	3.70-8.30	0.31	0.33-1.25	0.39	0.14-0.50	(-)
PID018	M	11Y	Unknown	Recurrent fever; Chronic EBV infection	168	300-600	845	1100-2200	37.1	62.328	59.6	100.128	2.77	4.6536	13.28[#]	6.09-12.85	0.36	0.67-2.01	2.2	0.52-2.16	(-)
PID019	M	2Y	Unknown	agammaglobulinemia	12	300-600	880	1500-2900	19.3	2.35074	80	9.6	0.57	0.0684	8.05	5.52-11.46	0.12	0.60-2.12	0.29	0.06-0.74	(-)
PID020	F	7Y	PIK3CD	Recurrent pulmonary infections; EBV infection	152	300-500	1052	1300-2200	63.5	96.52	30.5	46.36	5.01	7.6152	11.62[#]	6.09-12.85	3.81	0.67-2.01	1.32	0.52-2.16	(-)
PID021	F	3Y3M	Unknown	Recurrent pulmonary infections	935	300-600	2781	1900-2900	91.4	854.59	3.01	28.1435	2.84	26.554	6.92	4.95-12.74	11.53	0.65-2.01	0.44	0.33-1.89	(-)
PID022	M	10Y	Unknown	CVID	170	300-600	618	1100-2200	94.9	161.33	0.85	1.445	0.51	0.867	0.09	6.09-12.85	0.2	0.67-2.01	0.51	0.52-2.16	(-)
PID023	F	17Y	PIK3CD	Hyper IgM syndrome	52	300-500	820	1300-2200	17.1	8.892	17.1	8.892	14.5	7.54	5.81[#]	7.54-16.62	36.86	0.72-2.28	6.64	0.89-3.24	(-)
PID024	M	5Y7M	Unknown	agammaglobulinemia	176	300-600	1758	1500-2900	90	158.4	1.37	2.4112	2.62	4.6112	ND	4.95-12.74	0.04	0.65-2.01	0.08	0.33-1.89	(-)
PID025	M	1Y11M	Unknown	Recurrent fever	750	300-600	2572	1500-2900	94.3	707.25	1.56	11.7	1.42	10.65	8.15[#]	5.52-11.46	0.33	0.60-2.12	0.3	0.06-0.74	(-)
PID026	F	1Y5M	ADA2	Autoinflammatory; fever; skin rash	1192	300-600	1482	1500-2900	88.9	1059.688	2.63	31.3496	4.69	55.9048	9.96[#]	5.52-11.46	1.99	0.60-2.12	1.21	0.06-0.74	(-)
PID027	F	5M	Unknown	Agranulocytosis; inflammatory	725	300-800	2485	2800-3500	8.15	59.0875	0.47	3.4075	1.39	10.0775	7.47	3.70-8.30	0.9	0.33-1.25	0.36	0.14-0.50	(-)
PID028	M	5Y	Unknown	Eosinophilia	422	300-600	2478	1500-2900	84.1	354.902	4.91	20.7202	6.23	26.2906	14.39	4.95-12.74	1.14	0.65-2.01	2.09	0.33-1.89	(-)
PID029*	M	2M	Unknown	SCID	2.17	300-800	6.7	1800-3300	1.6	0.03472	44.8	0.97216	19.2	0.41664	4.15	3.70-8.30	0.05	0.33-1.25	0.03	0.14-0.50	(-)
PID030	M	5M	Unknown	Recurrent fever	1105	300-800	1346	1800-3300	97	1071.85	0.83	9.1715	0.69	7.6245	15.77	3.70-8.30	1.26	0.33-1.25	0.08	0.14-0.50	(-)
PID031	M	9M	Unknown	Recurrent severe infections	452	300-800	1956	1800-3300	93.7	423.524	2.45	11.074	1.68	7.5936	8.58	4.95-12.74	1.51	0.65-2.01	0.46	0.33-1.89	UD
PID032	M	1Y	BTK	XLA	1.9	300-600	4257	1500-2900	62.5	1.1875	28.1	0.5339	6.25	0.11875	ND	5.52-11.46	0.08	0.60-2.12	0.02	0.06-0.74	(-)
PID033	M	1Y	BTK	XLA	7	300-600	4965	1500-2900	93.6	6.552	5.32	0.3724	1.06	0.0742	9.41[#]	5.52-11.46	0.08	0.60-2.12	0.25	0.06-0.74	UD
PID034	M	2Y10M	Unknown	Recurrent infections	837	300-600	2268	1500-2900	90.4	756.648	6.25	52.3125	2.25	18.8325	27.1[#]	5.52-11.46	0.67	0.60-2.12	2.31	0.06-0.74	(-)
PID035*	F	5Y10M	Unknown	Recurrent infections	258	300-600	2688	1900-2900	59.5	153.51	12.3	31.734	18.3	47.214	14.39	4.95-12.74	1.34	0.65-2.01	2.9	0.33-1.89	UD
PID036	F	3Y7M	Unknown	Recurrent infections	379	300-600	1889	1900-2900	81.6	309.264	6.66	25.2414	6.79	25.7341	9.4	4.95-12.74	0.98	0.65-2.01	0.77	0.33-1.89	UD
PID037*	F	3M	RAG1	SCID	0.79	300-800	0.4	2800-3500	36.4	0.28756	36.4	0.28756	27.3	0.21567	7.75	3.70-8.30	0.06	0.33-1.25	0.04	0.14-0.50	UD
PID038	F	1M	IKBKG	Skin rash; Recurent infections	1507	300-800	3603	2800-3500	94.2	1419.594	1.78	26.8246	1.32	19.8924	8.2	3.70-8.30	1.25	0.33-1.25	0.33	0.14-0.50	UD
PID039	M	6Y	SH2D1A	XLP	920	300-600	3984	1500-2900	96.1	884.12	0.59	5.428	0.3	2.76	5.4[#]	4.95-12.74	0.12	0.65-2.01	0.18	0.33-1.89	UD
PID040	M	1Y3M	BTK	XLA	8.32	300-600	1836	1500-2900	89.6	7.45472	6.25	0.52	0	0	5.4[#]	5.52-11.46	0.06	0.60-2.12	0.12	0.06-0.74	UD
PID041	M	2M	Unknown	SCID	1082	300-600	11.3	1800-3300	97.7	1057.114	0.32	3.4624	0.034	0.36788	11.7[#]	3.70-8.30	0.18	0.33-1.25	0.04	0.14-0.50	UD
PID042	M	2Y11M	Unknown	Recurrent infections	756	300-600	2782	1500-2900	80	604.8	4.45	33.642	8.47	64.0332	7.47	5.52-11.46	1.15	0.60-2.12	0.47	0.06-0.74	UD
PID043	F	5M	RAG1	SCID	42	300-800	727	2800-3500	12.6	5.292	30.5	12.81	54.6	22.932	14.39[#]	3.70-8.30	1.42	0.33-1.25	0.27	0.14-0.50	UD
PID044	M	8Y	Unknown	Agammaglobulinemia;agranulocytosis	9.63	300-600	475.8	1100-2200	90.6	8.72478	3.12	0.300456	3.12	0.300456	4.9[#]	6.09-12.85	0.19	0.67-2.01	0.33	0.52-2.16	UD
PID045	M	5Y2M	CD40LG	Hyper IgM syndrome	473	300-600	2918	1500-2900	89.4	422.862	1.03	4.8719	1.56	7.3788	2.9	4.95-12.74	2.62	0.65-2.01	1.98	0.33-1.89	UD
PID046	F	5M	Unknown	Recurrent infections	43	300-800	407	2800-3500	90.9	39.087	7.27	3.1261	0	0	4.7	3.70-8.30	2.6	0.33-1.25	0.8	0.14-0.50	UD
PID047	M	5Y	Unknown	Fever; encephalopathy	435	300-600	426	1500-2900	86.4	375.84	2.94	12.789	5.67	24.6645	ND	4.95-12.74	0.04	0.65-2.01	0.18	0.33-1.89	UD
PID048	M	14Y	BTK	Hypogammaglobulinemia	1.38	300-500	1676.7	1100-2200	10	0.138	50	0.69	35	0.483	7.5[#]	6.98-14.26	0.03	0.56-2.16	0.55	0.92-2.50	UD
PID049	M	1Y11M	MHC II	Recurrent fever	572	300-600	4119	1500-2900	90.2	515.944	2.88	16.4736	3.88	22.1936	9.68[#]	5.52-11.46	3.57	0.60-2.12	0.22	0.06-0.74	UD
PID050	M	2M	WAS	WAS	487	300-800	921	2800-3500	96.4	469.468	1.25	6.0875	1.11	5.4057	8.85[#]	3.70-8.30	0.21	0.33-1.25	0.5	0.14-0.50	UD
PID051*	M	1Y9M	RAG1	Leaky SCID	16.64	300-600	179	1500-2900	38.8	6.45632	27.5	4.576	31.2	5.19168	10.51	5.52-11.46	1.6	0.60-2.12	1.92	0.06-0.74	UD
PID052*	F	5Y	AICDA	Hyper IgM syndrome	565	300-600	2146	1900-2900	3.46	19.549	50.1	283.065	44.5	251.425	0.01	4.95-12.74	15.4	0.65-2.01	0.33	0.33-1.89	UD

PID001 represents the patient of the RAG1 study. *, patients with high proportion (>10%) of DN B and memory B cells; #, after IVIG; ND, not detectable; UD, undetermined.
